# Quantifying Protein–Glycan Interactions Using Native Mass Spectrometry

**DOI:** 10.1002/mas.21943

**Published:** 2025-06-30

**Authors:** Duong T. Bui, Elena N. Kitova, Ling Han, Lara K. Mahal, John S. Klassen

**Affiliations:** ^1^ Department of Chemistry University of Alberta Edmonton Alberta Canada

**Keywords:** affinity, glycans, glycan‐binding proteins, native mass spectrometry, noncovalent interactions

## Abstract

Interactions between glycan‐binding proteins (GBPs) and carbohydrates (glycans) are essential to many biological processes relevant to human health and disease. For most GBPs, however, their glycan interactome—the repertoire of glycans recognized and their specificities—is poorly defined. The structural diversity of biologically relevant glycans and their limited availability in purified form, as well as their varied presentation, often as glycoconjugates, and weak affinities are key challenges hindering comprehensive glycan interaction mapping. Native mass spectrometry (nMS), a versatile, sensitive and label‐free tool for the discovery of GBP–glycan interactions and quantifying their stoichiometry and thermodynamic parameters, is poised to play a leading role in defining the glycan interactome of GBPs. Here, we review established nMS methodologies, as well as important experimental and instrumental considerations, for detecting GBP**–**glycan interactions in vitro, and reliably measuring their stoichiometry and affinity. Recent advances in nMS methods for high‐throughput library screening, including shotgun glycomics, and quantifying GBP interactions with glycoproteins and glycosphingolipids, are also described.

**Abbreviations**
ACE2: angiotensin‐converting enzyme 2
CaR: catch‐and‐release
CBM: carbohydrate‐binding module
CD: charge detection
CID: collision‐induced dissociation
COIN: concentration independent
CRD: carbohydrate recognition domain
CRM: charge residue model
CTB_5_
: cholera toxin B subunit homopentamer
CUPRA: competitive universal proxy receptor assay
ESI: electrospray ionization
FAC‐MS: frontal affinity chromatography coupled to MS
GBP: glycan binding protein
GL: glycolipid
GSL: glycosphingolipid
IS: internal standard
ITC: isothermal titration calorimetry
i.d.: inner diameters
*K*_a_
: association constant
*K*_d_
: dissociation constant
L: ligand
LC: liquid chromatography
MBL: mannose binding lectin
MEAN: MEmbrane ANchor‐assisted
m/z: mass‐to‐charge‐ratio
nanoESI: nanoflow ESI
ND: nanodisc
neoGLs: neoglycolipids
NMR: nuclear magnetic resonance
nMS: native mass spectrometry
NoV: norovirus
PD: picodisc
RBD: receptor binding domain
Ref: reference
*RF*
: response factor
RoR: ratio‐of‐the‐ratio
scFv: single chain variable fragment
SG: shotgun glycomics
Siglec: sialic‐acid‐binding immunoglobulin‐like lectin
SLOMO: slow mixing mode
SMALP: styrene‐maleic acid lipid particle.
SP: spike protein
SPR: surface plasmon resonance spectroscopy
STD: saturation transfer difference

## 1 Introduction

Carbohydrates (glycans) are an important class of biomolecules that, together with nucleic acids (DNA and RNA), peptides, proteins and lipids, are the building blocks of life and regulate all living processes. All cells possess a glycocalyx, a dense layer of complex glycans attached to peptides, proteins and lipids on the surface of the membrane (van Kooyk and Rabinovich 2008; Varki 2017). In mammals, the majority of secreted peptides and proteins are glycosylated (Varki 2017). Through their interactions with endogenous and exogenous glycan‐binding proteins (GBPs) or other receptors (e.g., other glycans and DNA and RNA), glycans mediate a wide range of physiological and pathophysiological processes, including cell adhesion, signal transduction, immune responses, diseases and viral, bacterial and fungal infections (Cagnoni et al. 2016; Clausen et al. 2020; Ge and Wang 2011; Imai and Kawaoka 2012; Li, Liu et al. 2021; Peters and Peters 2021; Prado Acosta and Lepenies 2019; Viswanathan et al. 2010). Identifying functional GBP–glycan interactions and characterizing their kinetic and thermodynamic parameters are critically important to understanding the biological roles played by glycans and may improve human health by guiding the development of diagnostics and therapeutics to detect and treat diseases (Rek et al. 2009).

Technological advances over the past 20 years have dramatically accelerated the discovery of GBP–glycan interactions and the characterization of their kinetic and thermodynamic parameters. However, the current understanding of the repertoire of glycans recognized by most GBPs remains far from complete. For example, the glycan binding properties of mannose‐binding lectin (MBL), a C‐type lectin involved in activating the lectin pathway of the complement system (part of the innate immune response to microorganisms), have resisted definition (Dommett et al. 2006). Similarly, the natural ligands of human sialic‐acid‐binding immunoglobulin‐like lectins (Siglecs), which are found on the surface of immune cells and regulate the immune response, remain to be fully elucidated (Rodrigues et al. 2020). The discovery and characterization of GBP–glycan interactions are inherently challenging due to: (1) the wide variety of naturally occurring glycans, with relatively few available in purified form, (2) the influence of presentation (e.g., as free glycans or glycoconjugates, such as glycolipids, glycopeptides or glycoproteins) and environment on glycan binding, and (3) the low affinities (*K*_d_ ∼1 mM) of most monovalent GBP–glycan interactions (Collins and Paulson 2004). It is estimated that there are > 10^4^ different glycan determinants in mammals (Cummings 2009). In contrast, the largest defined (purified) glycan libraries available for screening contain < 10% of these structures (Cummings 2009). The diversity of available structures can be expanded using natural libraries of glycans (derived from tissue, cells or biofluids). However, natural glycans are usually extracted as mixtures of hundreds of compounds, with concentrations spanning several orders of magnitude, and it is generally challenging to fully separate all components, particularly isomeric species. Most binding assays lack the specificity and resolution needed to analyze mixtures of glycans. Additionally, the unknown and unequal concentrations of glycans in natural libraries make it difficult to perform accurate quantitative screening to establish (relative) affinities. The aglycones of glycoconjugates add another layer to glycan diversity. Glycans may be conjugated to proteins, peptides, or lipids. Differences in the underlying structure, even when very subtle, can affect the binding properties of glycans (Somers et al. 2000). Finally, low‐affinity interactions can be challenging to detect with many established binding assays and require relatively large amounts of sample.

A variety of in‐solution and surface‐based assays are used to quantify the thermodynamic parameters of GBP–glycan binding (Dam et al. 2016; Huang et al. 2017; Wang et al. 2017). Available in‐solution assays include isothermal titration microcalorimetry (ITC) and nuclear magnetic resonance (NMR) spectroscopy. Although widely considered the “gold standard” for quantifying GBP–glycan interactions (Dam et al. 2016; Nagae, Ikeda et al. 2013), ITC measurements generally require large amounts of purified GBP and glycan ligand (typically 0.1–1.0 mg). Moreover, a substantial enthalpy change (∆*H*) accompanying ligand binding is needed, and, as a result, ITC often cannot be used for low‐affinity interactions (Turnbull and Daranas 2003). In addition, as ITC lacks specificity, it is normally restricted to individual GBP–glycan interactions and is not well suited for systems involving multiple binding equilibria. Saturation transfer difference (STD) and imaging STD NMR spectroscopy have been used to measure *K*_d_ for a variety of GBP–glycan complexes (Hanashima et al. 2015; Nagae, Yamanaka et al. 2013). However, NMR methods require a large amount of sample (~mg of GBP and glycan) (Creutznacher et al. 2021), and the reliability of the methods for measuring low‐affinity interactions is questionable (Han et al. 2018). The most widely used surface‐based assay for quantifying the kinetics of GBP–glycan binding, from which *K*_d_ values are deduced, is surface plasmon resonance (SPR) spectroscopy (Wang et al. 2017; Wesener et al. 2015). However, as with other surface‐based assays, the immobilization of the glycan (or GBP) on the sensor chip may lead to nonnative and nonaccessible conformations/orientations, which affect the measured binding properties (Chiodi et al. 2022). Additionally, immobilization of one of the binding partners alters the entropy change (∆*S*) of the interaction and, consequently, influences the *K*_d_ value measured (Chiodi et al. 2022). Increasingly, bio‐layer interferometry (BLI), a dipping biosensor, is being used to study GBP‐glycan interactions. As BLI doesn't require a sensor chip, the technique is more versatile than SPR spectroscopy and provides higher sample throughput potential (Ji and Woods 2018).

High‐throughput screening of defined and natural libraries of glycans drives ligand discovery. Glycan microarray‐based screening dominates the ligand discovery landscape and provides a convenient approach for rapidly surveying glycan specificities (Gao et al. 2019; Muthana 2020; Rillahan and Paulson 2011). Glycan microarrays are typically constructed from libraries of purified, modified glycans, which are immobilized (either physically or chemically) on a surface. Interactions with a target GBP, following washing steps, are detected using a fluorescence readout (Gao et al. 2019). Ideally, the relative fluorescence signal reflects the relative binding strength and, thereby, provides insight into glycan binding specificity (Gao et al. 2019). However, while this technology has greatly accelerated discovery, it is, at best, semiquantitative and suffers from numerous limitations, including deleterious effects of glycan labeling and immobilization on binding and a high false negative rate for weak interactions due to the washing steps (Temme et al. 2019). Frontal affinity chromatography coupled to MS (FAC‐MS) has also been used for quantitative screening of glycan libraries against target GBPs. In FAC‐MS, the GBPs are immobilized on the stationary phase, while the glycans are in the mobile phase. The retention time (elution volume), column binding capacity and ligand concentration are used for affinity calculation (Iwaki and Hirabayashi 2018; Kasai 2021; Zhang et al. 2001). Although FAC‐MS affords quantitative, high‐throughput screening, immobilization may alter the GBP binding properties. Moreover, the assay has relatively large sample requirements, particularly for the detection of low‐affinity interactions (Nakamura‐Tsuruta et al. 2007).

Over the last decade, native MS (nMS)—which typically involves electrospray ionization (ESI)‐MS implemented under native‐like solution conditions and experimental and instrumental parameters that preserve the noncovalent interactions present in solution during analysis—has emerged as a highly versatile, label‐ and immobilization‐free method for the discovery and quantification of GBP**–**glycan interactions (El‐Hawiet et al. 2013; Kitova et al. 2012; Park et al. 2020). nMS not only provides a direct readout of binding stoichiometry but can also quantify multiple equilibria simultaneously, including coupled equilibria (involving the same or different ligands), and can directly establish positive cooperativity (Lin et al. 2014). Absolute *K*_d_, in the range of 10 mM–0.1 µM, have been measured for glycan ligand binding to a wide variety of GBPs, including lectins, antibodies, enzymes, and viral proteins (Han et al. 2018; Kitova et al. 2001; Shams‐Ud‐Doha et al. 2017).

In recognition of the important and ever‐expanding role of nMS in functional glycomics—the discovery of biologically relevant glycan ligands of GBPs and characterization of their binding properties—this review describes established nMS methodologies, as well as important experimental design considerations, for detecting GBP**–**glycan interactions and reliably measuring their stoichiometry and affinity. Recent advances in nMS methods for quantifying GBP interactions with glycoconjugates and high‐throughput library screening, including shotgun glycomics, are also described.

## 2 Fundamentals of nMS Quantification of GBP–Glycan Binding

### 2.1 Direct nMS Analysis

**Figure 1:**
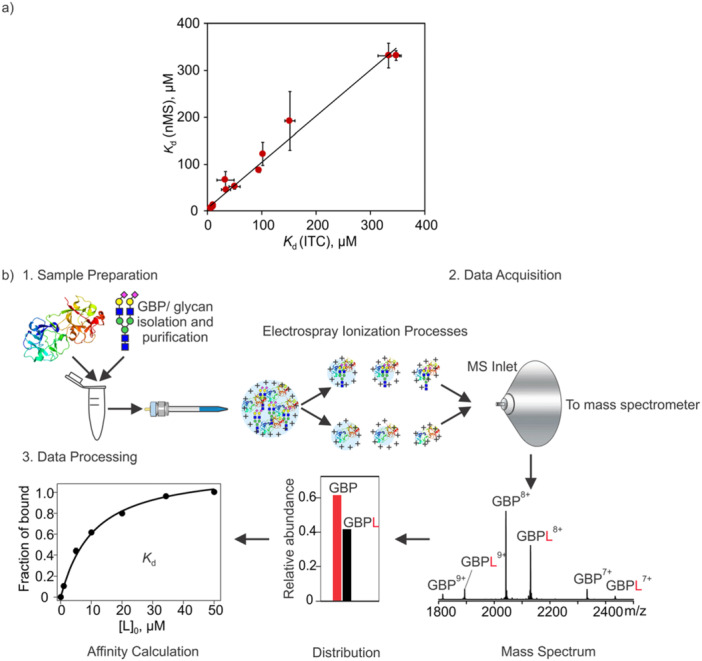
(a) Comparison of *K*_d_ values for GBP**–**glycan interactions measured by nMS and ITC (Bachhawat‐Sikder et al. 2001; Báez Bolivar et al. 2021; Diehl et al. 2010; López‐Lucendo et al. 2004; Noll et al. 2016; Shams‐Ud‐Doha et al. 2017; Sun et al. 2007; Wang et al. 2003; Yao et al. 2014). (b) Overview of the main steps of the nMS assay for quantifying GBP**–**glycan ligand (L) binding illustrated for a 1:1 complex (GBPL). Step 1, sample preparation: a solution of GBP and the L is prepared at the desired initial concentrations and an aliquot loaded into a (nano)ESI emitter. Step 2, data acquisition: GBP and GBPL complex ions are transferred from solution to the gas phase by (nano)ESI and the gaseous ions are sampled into the mass spectrometer and detected. Step 3, data analysis: the abundance ratio of GBPL to GBP ions is determined from the mass spectrum. The *K*_d_ can be calculated from the initial concentrations and the abundance ratio from single measurement. To reduce uncertainty, *K*_d_ can be determined using a titration approach and by applying nonlinear fitting. [Color figure can be viewed at wileyonlinelibrary.com]

Quantifying the binding stoichiometry and affinity of GBP**–**glycan interactions by direct nMS analysis relies on transferring, intact, the GBP**–**glycan complexes from solution to the gas phase by ESI (or related method) and preserving their relative distribution throughout MS analysis, such that the mass spectrum provides a snapshot of solution composition. Ganem and coworkers were the first to demonstrate (using enzyme‐substrate and enzyme‐product complexes) that biomolecular interactions can survive the ESI process and be detected by MS (Ganem et al. 1991a). The first determination of protein–ligand affinity by nMS was demonstrated by Loo and co‐workers in 1993 (Loo et al. 1993). In 2001, Klassen and co‐workers described the quantification of GBP**–**glycan complexes by nMS for the first time (Kitova et al. 2001). The nMS assay has since been rigorously tested and shown to produce *K*_d_ values that are in good agreement with other in‐solution assays, such as ITC (Figure 1a), when performed under appropriate experimental and instrumental conditions.

The general workflow for direct nMS quantification of a 1:1 GBP**–**glycan complex is outlined in Figure 1b. The main steps are: (1) solution preparation, wherein purified GBP and glycan ligand (L) are mixed at concentrations that produce detectable levels of complex, (2) transfer of free and L**‐**bound GBP species from solution to the gas phase by ESI and mass spectrum acquisition, and (3) analysis of the relative abundance of free and L**‐**bound GBP species for *K*_d_ calculation. nMS affinity measurements can, in principle, be carried out using conventional (pump‐driven) ESI. However, due to the high sample requirements of ESI and the potential for nonspecific adduct formation, affinity measurements are normally implemented using nanoflow ESI (nanoESI). NanoESI is typically performed using glass (borosilicate) capillaries pulled to a small diameter (typically internal diameter (i.d.) is in the range of 1–3 µm) at one end (Nguyen et al. 2019; Schmidt et al. 2003; Susa et al. 2017a). Because of the low solution flow rates (usually from 10 nL min^−1^ to 100 nL min^−1^), only a few μL of sample solution are needed for nMS analysis (Schmidt et al. 2003). Moreover, nanoESI produces droplets with diameters that are much smaller (<μm) than conventional ESI, which reduces the probability of nonspecific complex (adduct) formation and the need for thermal/collisional heating to produce fully desolvated ions (Schmidt et al. 2003). Appropriate GBP and glycan concentrations for nMS affinity measurements are system‐dependent. Typical concentrations needed to detect GBPs (and proteins in general) are in the 1–10 µM range. Glycan ligand concentrations are selected according to the affinities of the interactions and are generally between 0.1 µM and 10 mM. For weak (~mM) interactions, it is recommended to use a titration approach, vide infra.

In principle, any ESI‐MS instrument can be used for nMS‐based *K*_d_ measurements and, indeed, GBP–glycan affinities have been acquired using a variety of MS platforms (Báez Bolivar et al. 2021; El‐Hawiet et al. 2013; Liu, Michelsen et al. 2012; Wang et al. 2003). However, the analysis of low‐affinity interactions and those involving high MW (hundreds of kDa to MDa) or highly heterogeneous GBPs is challenging. Such measurements generally require highly efficient ion transmission of the GBP and GBP−glycan complex ions and, often, high mass analyzer resolution. The introduction of the Ultra High Mass Range (UHMR) Q‐Exactive Orbitrap mass spectrometer (Thermo Fisher Scientific, Bremen, Germany) has had a notable impact on the characterization of biomolecular complexes, including GBP−glycan interactions, by nMS. The instrument affords high resolution capabilities (200,000 at mass‐to‐charge‐ratio (m/z) 400) over a wide mass range (m/z from 350 to 80,000; 150 to 80,000 with a special licence), provides efficient transmission of high MW GBPs and their complexes, and possesses a high dynamic range (> 5000:1), which facilitates the detection and quantification of low‐affinity complexes.

#### 2.1.1 Measuring a Single Binding Equilibrium

In most nMS‐based studies of GBP–glycan binding, it is assumed that the measured relative abundances (*Ab*) of free and L‐bound GBP ions accurately reflect their relative concentrations in solution. Under this assumption, for a 1:1 GBP–glycan complex (Equation 1a), the measured (apparent) total ion *Ab* ratio of GBPL to GBP (*R*_app_) is taken to be equivalent to the concentration ratio (*R*, Equation 1b) (Bennett et al. 2022; Kitova et al. 2012). The corresponding *K*_d_, or the association constant (*K*_a_ = 1/*K*_d_), can then be calculated from *R* (≡ *R*_app_) and the initial concentrations of glycan ([L]₀) and protein ([GBP]₀) using Equation (1c):

(1a) GBPL⇌GBP+L

(1b) $$R_{\mathrm{app}}=\frac{\mathrm{Ab}\left(\text{GBPL}\right)}{\mathrm{Ab}\left(\text{GBP}\right)}\approx \frac{\left[\text{GBPL}\right]}{\left[\text{GBP}\right]}=R$$

(1c) $$K_{d}=\frac{\left[\text{GBP}\right]\left[L\right]}{\left[\text{GBPL}\right]}=\frac{{\left[L\right]}_{0}}{R}-\frac{{\left[\text{GBP}\right]}_{0}}{R+1}$$

A distinctive advantage of nMS‐based quantification of GBP–glycan binding (and biomolecular interactions in general) is that *K*_d_ can, in principle, be calculated from a single mass spectrum acquired at a specific concentration of GBP and glycan. However, for greater accuracy, *K*_d_ is typically determined as the average value from multiple measurements conducted at different initial concentrations. For low‐affinity interactions (~mM), where the ion signal of the GBP–glycan complex is often weak, it is generally advisable to use a titration approach to reduce uncertainty. To implement the titration approach, nMS is performed on a series of solutions with fixed [GBP]_0_ and varying [L]_0_. The *K*_d_ for a 1:1 binding equilibrium is obtained by nonlinear fitting of Equation (2a) to the fraction of ligand‐bound GBP (*R*/(*R* + 1)) measured by nMS:

(2a) $$\frac{R}{R+1}=\frac{{\left[\mathrm{GBP}\right]}_{0}+{\left[L\right]}_{0}+K_{d}-\sqrt{{\left(K_{d}-{\left[L\right]}_{0}+{\left[\mathrm{GBP}\right]}_{0}\right)}^{2}+4K_{d}{\left[L\right]}_{0}}}{2{\left[\mathrm{GBP}\right]}_{0}}$$

The titration approach is also useful for studying multisubunit GBPs, where the number of binding sites may be unknown, and, as described below, for identifying and correcting for in‐source dissociation (Báez Bolivar et al. 2021).

While no longer widely employed, the Scatchard method (Scatchard 1949), which linearizes binding data, can also be used to assess *K*_d_. In this approach, the ratio of [GBPL]/([L][GBP]_0_) is plotted against the ratio of [GBPL]/[GBP]_0_, Equation (2b):

(2b) $$\frac{\left[\mathrm{GBPL}\right]}{{[L]\left[\text{GBP}\right]}_{0}}=\frac{1}{K_{d}}-\frac{\left[\mathrm{GBPL}\right]}{{\left[\text{GBP}\right]}_{0}}\frac{1}{K_{d}}$$

This expression can be rewritten in terms of the fraction of ligand‐bound protein, Equation (2c):

(2c) $$\frac{R}{\left(R+1\right)}\frac{1}{\left[L\right]}=-\frac{R}{R+1}\frac{1}{K_{d}}+\frac{1}{K_{d}}$$

The magnitude of *K*_d_ can be found from linear regression of a plot of *R*/(*R* + 1) versus [L] (Dennhart and Letzel 2006; Lim et al. 1995; Loo et al. 1997).

**Figure 2:**
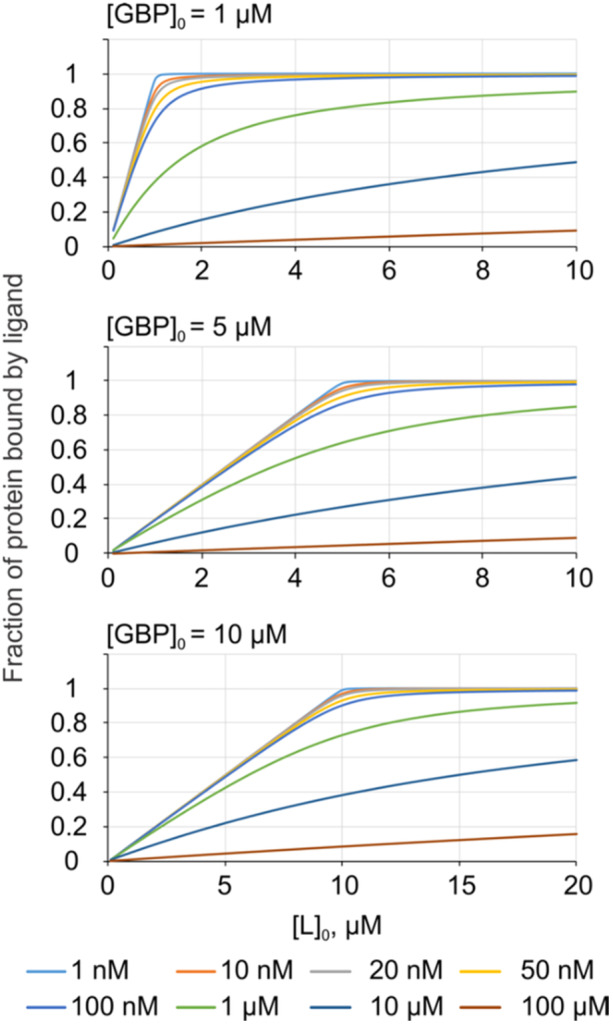
Theoretical titration curves for high‐affinity GBP**–**glycan interactions at different initial concentrations of GBP ([GBP]_0_). Solid curves represent different *K*_d_ values, ranging from 1 nM to 100 µM. [Color figure can be viewed at wileyonlinelibrary.com]

Native MS affinity measurements are usually performed using a [GBP]_0_ in the µM range, which generally provides a sufficient signal‐to‐noise ratio (S/N). Shown in Figure 2 are theoretical titration curves calculated for a [GBP]_0_ of 1, 5, and 10 µM and different *K*_d_. At higher affinity and higher [GBP]_0_, the fraction of GBP bound to ligand increases stoichiometrically with ligand concentration, producing titration curves that can't be distinguished. For example, at [GBP]_0_ of 1 µM, the titration curves corresponding to *K*_d_ ≤ 20 nM are indistinguishable, within experimental errors. At [GBP]_0_ of 10 µM, curves corresponding to *K*_d_ ≤ 100 nM are indistinguishable. Therefore, for high affinity interactions, it is important to choose a suitable [GBP]_0_ (≤ 10 *K*_d_).

#### 2.1.2 Measuring Multiple Binding Equilibria

A unique feature of the nMS assay is the ability to quantitatively monitor multiple equilibria simultaneously, whether they involve independent or coupled interactions. For independent equilibria, nMS data analysis follows the procedures outlined in Section 2.1.1. For coupled equilibria, explicit consideration of mass balance is required. Two common coupled equilibria scenarios amenable to nMS analysis are: (1) sequential binding of a common L to a GBP with multiple binding sites and (2) binding of multiple distinct ligands (e.g., from a library) to a GBP with one or more binding sites. A mathematical framework for analyzing nMS data for both cases is provided below.

##### 2.1.2.1 Sequential Glycan Ligand Binding to a GBP

Many GBPs possess multiple glycan binding sites (Dam and Brewer 2010; Lee and Lee 2000) and, through multivalent binding (simultaneous engaging multiple glycan ligands associated with a cell, particle or glycoconjugate), achieve higher functional affinity (avidity). Native MS, which enables the simultaneous monitoring of multiple interacting species and their complexes, is ideally suited for quantifying sequential ligand binding and establishing the equivalence and independence of the binding sites. In the case of a GBP possessing *q* binding sites, the dissociation constant (*K*_d,*i*_) for the successive unbinding steps (Equations 3a) can be described by Equation (4a):

(3a) $$\begin{array}{c} K_{d,1}\\ \text{GBPL}⇌\text{GBP}+L \end{array}$$

(3b) $$\begin{array}{c} K_{d,2}\\ {\text{GBPL}}_{2}⇌\text{GBPL}+L \end{array}$$

(3c) $$\begin{array}{c} K_{d,i}\\ {\text{GBPL}}_{i}⇌{\text{GBPL}}_{i-1}+L \end{array}$$

(4a) $$K_{d,i}=\frac{\left.R_{i-1}{\left([,L,]\right.}_{0}-\frac{\left(R_{1}+2R_{2}+\ldots +qR_{q}\right){\left[\mathrm{GBP}\right]}_{0}}{1+R_{1}+R_{2}+\ldots +R_{q}}\right)}{R_{i}}$$

where *i* is the number of occupied binding sites (0 < *i* ≤ *q*), and *R*_i_ can be derived from Equation (1b) for each L‐bound species.

In cases where the binding sites are equivalent (identical) and independent, the microscopic (intrinsic, per binding site) *K*_d_ (*K*_d,intrin_) and macroscopic *K*_d,*i*_ values are related through statistical factors that reflect the number of occupied and unoccupied sites, Equation (4b):

(4b) $$K_{d,\text{intrin}}=\frac{\left(q-i+1\right)K_{d,i}}{i}$$

As such, nMS measurements (of *q* and *K*_d,_ *_i_ d*) provide a direct approach to establish whether binding sites are equivalent and independent. Several applications of nMS to study sequential glycan binding to GBPs with equivalent and independent binding sites has been reported: Shiga‐like toxin B subunit homopentamer (Kitova et al. 2001), human galectin‐7 homodimer (Shams‐Ud‐Doha et al. 2017) and P‐dimer of the Saga strain of norovirus (Báez Bolivar et al. 2021; Han et al. 2018). Deviation from Equation 4b will arise when the binding sites are: (i) equivalent and dependent, (ii) nonequivalent and independent and (iii) nonequivalent and dependent. When the binding sites are equivalent but dependent (Badjić et al. 2005; Errington et al. 2019; Lopez‐Fontal et al. 2016), ligand binding at one site will increase (positive cooperativity) or decrease (negative cooperativity) the affinity of interactions at other sites. In the case of cooperative binding, the differences in *K*_d,*i*_ values will reflect *K*_d, intrin_ and both the statistical factors and cooperativity factors (*α*_*i*_), Equation (5):

(5) $$K_{d,\;\text{intrin}}=\alpha_{i}\frac{\left(q-i+1\right)K_{d,i}}{i}$$

Thus, if the binding sites are known to be equivalent, then *α*_*i*_ can be directly determined from the measured *K*_d,*i*_. However, negative cooperativity is indistinguishable from the situation where the binding sites are nonequivalent and independent. Moreover, it may not be possible to directly establish positive cooperativity from the measured *K*_d,*i*_ in the case of nonequivalent binding sites.

The first use of nMS to identify and quantify positive cooperativity in GBP–glycan binding involved the GM1 ganglioside pentasaccharide (GM1_os_) and its interactions with the cholera toxin B subunit homopentamer (CTB_5_), which possesses five equivalent (primary) binding sites (Lin et al. 2014). The systematic decrease in measured *K*_d,i_ showed unambiguously that GM1_os_ binding to CTB_5_ exhibits positive cooperativity, consistent with the findings of Homans and coworkers (Turnbull et al. 2004). By applying their binding model, wherein cooperativity increases with the number of ligand‐occupied neighboring subunits, cooperativity factors of 0.6 (1/1.7, one neighboring subunit occupied) and 0.3 (1/2.9, two neighboring subunits occupied) were obtained from the nMS data (Lin et al. 2014). Notably, these values agree with cooperativity factors (0.6 (1/1.9) and 0.3 (1/3.4) respectively) deduced from ITC data (Turnbull et al. 2004).

**Figure 3:**
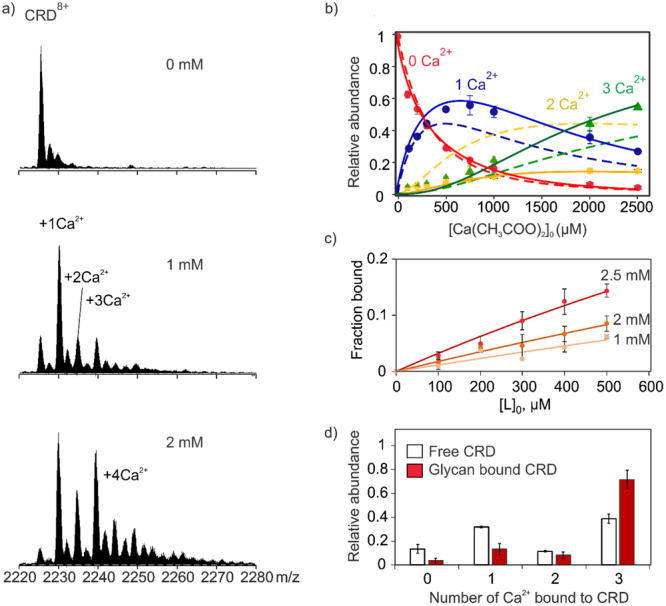
Identifying cooperative Ca^2+^ binding to DC‐SIGN CRD by nMS. (a) Region of the mass spectra corresponding to the +8 charge state of the CRD measured at different concentrations (0 mM, 1.0 mM, and 2.0 mM) of Ca(CH_3_COOH)_2_. (b) Two different binding models were used to predict the relative abundances of different species of Ca^2+^‐bound CRD (experimental values shown as solid symbols): the dashed lines correspond to a model assuming three independent and equivalent binding sites, the solid lines correspond to a model assuming three equivalent but dependent binding sites with cooperativity factors of 6 (α1) and 0.25 (α2). (c) Influence of Ca(CH_3_COOH)_2_ concentration (1.0, 2.0, 2.5 mM) on CRD‐glycan (L) interactions (d). Distribution of Ca^2+^ bound to DC‐SIGN CRD (white) and CRD‐L (red) in the aqueous ammonium acetate solutions (200 mM, pH 7.4) of CRD (1.0 μM), L (500 μM), and Ca(CH_3_COOH)_2_ 1.0 mM. Figures were adapted from published data (Báez Bolivar et al. 2021). [Color figure can be viewed at wileyonlinelibrary.com]

Native MS measurements also uncovered cooperativity in the sequential binding of Ca^2+^ to the carbohydrate recognition domain (CRD) of the dendritic cell‐specific intercellular adhesion molecule‐3‐grabbing nonintegrin (DC‐SIGN) immune lectin (Báez Bolivar et al. 2021). The CRD possesses three glycan binding sites and, although binding at each site is Ca^2+^ dependent, the thermodynamics of Ca^2+^ binding and the roles that Ca^2+^ plays in glycan binding are not fully elucidated (Guo et al. 2004; Valverde et al. 2019). Native MS measurements performed on solutions of DC‐SIGN CRD containing Ca(CH_3_COO)_2_, at concentrations up to 2 mM, identified sequential Ca^2+^ binding to the CRD (Figure 3a). After correction of the mass spectra for nonspecific binding using the reference protein method (see Section 2.2.4), the nMS data showed that up to three Ca^2+^ bind specifically to the CRD. Notably, the concentration‐dependent relative abundances of Ca^2+^‐bound CRD species measured experimentally (Figure 3b) are inconsistent with values predicted from a model that treats the three Ca^2+^ binding sites as equivalent and independent (Figure 3b). Instead, the data are well described by a model that treats the binding sites as equivalent but dependent (with cooperativity factors of 6 (α1) and 0.25 (α2)) (Figure 3b). Interestingly, while Ca^2+^ attachment enhanced glycan binding, with *K*_d_ values decreasing with increasing Ca^2+^ concentration (Figure 3c), glycan binding was also found to stabilize the CRD‐Ca^2+^ interactions (Figure 3d) (Báez Bolivar et al. 2021). Such synergistic binding effects would be difficult to identify with any other assay.

##### 2.1.2.2 Competitive Glycan Ligand Binding to a GBP

nMS can simultaneously monitor interactions between a GBP and multiple ligands of distinct MWs. In the case of a GBP possessing a single binding site, the equilibria can be generally described by Equation (6a):

(6a) $$\begin{array}{c} K_{d,1}\\ {\text{GBPL}}_{1}⇌\text{GBP}+L_{1} \end{array}$$

(6b) $$\begin{array}{c} K_{d,2}\\ {\text{GBPL}}_{2}⇌\text{GBP}+L_{2} \end{array}$$

(6c) $$\begin{array}{c} K_{d,x}\\ {\text{GBPL}}_{x}⇌\text{GBP}+L_{x} \end{array}$$

In principle, *K*_d_ for each ligand (*K*_d,*j*_) present in a mixture of *x* ligands (1 ≤ *j* ≤ *x*) can determined from a single measurement, Equation (6d):

(6d) $$K_{d,j}=\frac{{\left[L_{j}\right]}_{0}-\frac{R_{j}{\left[\mathrm{GBP}\right]}_{0}}{1+R_{1}+R_{2}+\ldots +R_{x}}}{R_{j}}$$

where *R*_*j*_ is the ratio of total *Ab* of GBP bound to L_*j*_ over the *Ab* of free GBP (Equation 1b).

One of the earliest demonstrations of this approach involved establishing the binding of antithrombin to a heterogeneous mixture of heparin oligosaccharides to antithrombin (Abzalimov et al. 2007). Examples of quantitative glycan library screening have also been reported. For instance, nMS was used to screen defined libraries of over 200 oligosaccharides against a single‐chain fragment (scFv) of a monoclonal antibody (mAb) Se155‐4 and the antigen‐binding fragment (Fab) of a mAb (CS35) targeting *Mycobacterium tuberculosis* (El‐Hawiet, Shoemaker et al. 2012). However, when ligands have similar MWs, resolving their bound forms (to the GBP) can be challenging. For example, to distinguish the human milk oligosaccharides (HMOs) Fuc₂GalGlcNAcGalGlc (999.36 Da) and Neu5AcGalGlcNAcGalGlc (998.34 Da) binding to GBP with MWs ~30 kDa (e.g., human galectin 1), a resolving power of at ~30,000 is required (Shams‐Ud‐Doha et al. 2017). For larger and/or glycosylated GBPs, even higher resolving powers are required, which exceed the capabilities of many mass analyzers (Zubarev and Makarov 2013). In such cases, the Catch‐and‐Release (CaR) approach (described in Section 4) is preferred for high‐throughput screening of glycan libraries against GBPs.

##### 2.1.2.3 Glycan Ligand Binding to Multiple GBPS. Ratio‐of‐the‐Ratio (ROR) Method

Measuring glycan ligand binding to multiple GBPs in the same solution using nMS is a powerful method for assessing ligand selectivity and accurately establishing differences in ligand affinity. In a solution containing *x* GBPs (GBP_*j*_, 1 ≤* j* ≤ *x*), each with an affinity for L of *K*_d,*j*_, the coupled equilibria can be described by Equation (7a):

(7a) $$\begin{array}{c} K_{d,1}\\ {\text{GBP}}_{1}L⇌{\text{GBP}}_{1}+L \end{array}$$

(7b) $$\begin{array}{c} K_{d,2}\\ {\text{GBP}}_{2}L⇌{\text{GBP}}_{2}+L \end{array}$$

(7c) $$\begin{array}{c} K_{d,x}\\ {\text{GBP}}_{x}L⇌{\text{GBP}}_{x}+L \end{array}$$

Notably, the ratio of *K*_d,*j*_ for two different GBPs, for example GBP_*j*_ and GBP_1_, is equal to the corresponding ratio of *R*_1_ to *R*_*j*_ (Equation 1b), as shown in Equation (8a:)

(8a) $$\frac{K_{d,j}}{K_{d,1}}=\frac{{\left[L\right]}_{0}-\frac{R_{j}}{1+R_{j}}{{[\text{GBP}}_{j}]}_{0}}{R_{j}}\frac{R_{1}}{{\left[L\right]}_{0}-\frac{R_{1}}{1+R_{1}}{{[\text{GBP}}_{1}]}_{0}}$$

(8b) $$\frac{K_{d,j}}{K_{d,1}}=\frac{\left[L\right]R_{1}}{R_{j}\left[L\right]}=\frac{R_{1}}{R_{j}}$$

This approach, known as the RoR method, is independent of the concentrations of GBPs and ligand used, thus enabling highly precise determination of relative *K*_d_. It is particularly well‐suited for discerning small differences in *K*_d_ or when relative *K*_d_ values of individual GBPs in a mixture are of interest.

Williams and Kirsch (Krishnaswamy et al. 2006) were among the first to describe the RoR method and demonstrated it for the determination of the relative *K*_d_ of a series of high‐affinity protein‐protein interactions. The first application of RoR to GBP–glycan binding involved a comparison of the affinities of HMOs for human galectin‐3 (GAL‐3) proteoforms and the C‐terminal domain of GAL‐3 (GAL‐3C) (Shams‐Ud‐Doha et al. 2017). More recently, it was used to evaluate the effect of fluorophore‐labeling on the glycan binding properties of GAL‐3C (Kitova et al. 2019) and to assess relative affinities of hemoglobin for asialo‐haptoglobin (Wu et al. 2018).

### 2.2 Practical Challenges to Quantifying GBP–Glycan Interactions by nMS

The reliable quantification of *K*_d_ for GBP–glycan interactions by nMS requires that the measured relative *Ab* of GBP and GBPL ions reflect their relative concentrations in solution. However, there are several potential sources of error, and these need to be carefully controlled if meaningful affinity data is to be obtained. The most significant sources of error are: (1) ESI‐associated electrochemical reactions that change solution composition, (2) differences in nMS response factors (*RF*), (3) in‐source dissociation, and (4) nonspecific complex formation. A brief overview of these sources of error and mitigation strategies are provided below.

#### 2.2.1 ESI‐Induced Electrochemical Changes in Solution Composition

The buffer used in nMS serves a dual purpose: controlling pH and ionic strength and maintaining charge balance during the ESI process (Leney and Heck 2017). Ammonium acetate, a volatile salt, is most commonly used (Ganem et al. 1991b, 1991a; Katta and Chait 1991). Ions will move under the influence of the applied electric field; the direction depends on their charge polarity and the direction of the field. Accumulation of ions at the surface of the solution at end of the ESI emitter leads to the ejection of charged droplets. Because the droplets carry away charge, electrochemical reactions at the ESI electrode occur to maintain charge neutrality. In aqueous ammonium acetate solutions, the dominant reactions involve oxidation or reduction of H₂O, producing H₃O⁺ (positive ion mode) and OH^−^ (negative ion mode) (Van Berkel et al. 2001). These electrochemistry products alter the pH of the solution and, potentially, induce changes in the structures and interactions of GBPs (Wang et al. 2003). The change in pH in the emitter can exceed 2 units h^−1^ when performing nanoESI using weakly buffered solutions (Wang et al. 2003). Although ammonium acetate has a low buffer capacity in the neutral pH range, the use of high concentrations (≥ 100 mM) can guard against deleterious pH changes (Wang et al. 2003). For experiments requiring very long acquisition times (> 1 h), ammonium acetate concentrations > 200 mM are advised (Li et al. 2023, 2020, Li, Kitov et al. 2021; Bui et al. 2022b; Bui et al. 2023). While alternatives to ammonium acetate, such as fluorinated amines, have been proposed to achieve better buffering capacity in the physiological pH range (Davis et al. 2023), ammonium acetate remains the salt (buffer) of choice for most nMS experiments.

#### 2.2.2 Nonuniform Response Factors (*RF*s)

The assumption that *R*_app_ is equivalent to *R* (Equation 1b) is valid only in cases where the GBPL complex and GBP ions have similar *RF*s and the binding equilibrium is not perturbed by the ESI process or MS analysis. Generally, the abundances of GBP and GBPL ions are related to their solution concentrations through their *RF*s (*RF*_GBP_ and *RF*_GBPL_, respectively), Equation (19a):

(9a) $$\mathrm{Ab}\left(\text{GBP}\right)={\mathrm{RF}}_{\text{GBP}}\left[\text{GBP}\right]$$

(9b) $$\mathrm{Ab}\left(\text{GBPL}\right)={\mathrm{RF}}_{\text{GBPL}}\left[\text{GBPL}\right]$$

The *RF* of a given analyte reflects the ionization (ion formation), transmission, and detection efficiencies. The relative ionization efficiency depends on the physical properties of the analyte (such as size, hydrophobicity, surface activity, ion solvation energies, and gas‐phase acidity/basicity), spray potential, solution composition and ionic strength (Sjöberg et al. 2001). The presence of other ions in the solution, especially at high concentrations, may also influence analyte *RF* due to competition for surface charge (Tang et al. 2004). The type of mass spectrometer used and the specific instrumental settings, including source conditions and resolution, also strongly affect the relative abundance of detected species. From a comparison of GBP–glycan binding data acquired using nMS and ITC, it was shown that GBP and GBPL ions tend to exhibit similar *RF*s when L has a MW less than ~5% that of the GBP (Lin et al. 2013). This condition is satisfied for most GBP–glycan complexes involving free oligosaccharides. However, for interactions involving polysaccharides, glycoproteins or glycolipids (GLs) presented in model membranes, the differences in *RFs* can be significant (Bui et al. 2022b, 2023). Since nonuniform *RF*s are more common than not, correcting for these differences is essential for accurate quantification. Without *RF* correction, the measured *K*_d_ values may be in error by more than two orders of magnitude (Bui et al. 2022b, 2023, 2024).

Various methods have been developed to account for nonuniform *RFs* in nMS affinity measurements. Gabelica and co‐workers demonstrated a technique using a nonreactive internal standard (IS) that closely resembles one of the interacting species, combined with mass balance considerations, to quantify ligand affinities for DNA duplexes (Gabelica et al. 2009). By conducting measurements at different concentrations, a set of independent linear equations is generated, which can be solved to determine the relative *RF* of each complex formed and the associated *K*_d_. This approach, however, requires a suitable IS, which is often not available. Gross and co‐workers introduced a mathematical model that accounts for both the relative *RF* and extent of in‐source dissociation (of complexes) to quantify receptor‐ligand binding and oligomerization reactions (Chitta et al. 2005; Wilcox et al. 2008). The *K*_d_ and relative *RF* are established by numerically solving a system of differential equations that describe the abundances of the interacting species. Zenobi and colleagues demonstrated a technique to simultaneously determine *K*_d_ and relative *RF* for protein and DNA dimerization reactions based on global fitting of titration data (Barylyuk et al. 2013; Boeri Erba et al. 2011; Root et al. 2017). These approaches, however, requires high‐quality data with minimal scattering to obtain reliable parameters. Moreover, the relative RF must be constant over the range of concentrations analyzed. These conditions can often be met using a pump‐driven ESI source. However, quantification of biomolecular interactions is usually carried out using single use nanoESI emitters and variations in geometry lead to tip‐to‐tip variations in relative *RF*s. Consequently, the use of multiple emitters to generate a titration curve can be a significant source of error.

**Figure 4:**
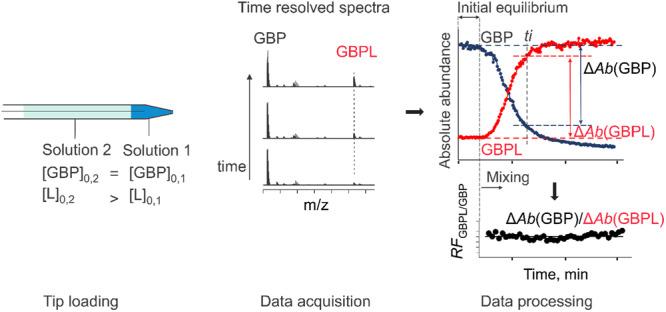
Schematic overview of the SLOMO‐nMS assay used to determine *K*_d_ for glycan ligand (L) binding to GBP. The nanoESI emitter is loaded with two different solutions (Solutions 1 and 2) then time‐resolved data is collected. Before the onset of mixing at the end of the emitter, *R*_app_ corresponding to the initial equilibrium establish in Solution 1 with known GBP and GBPL concentrations is measured. Once mixing takes place, the changes in the abundances of the GBP and GBPL (Δ*Ab*(GBP) and Δ*Ab*(GBPL). respectively) are used to calculate *RF*_GBP/GBPL_ at specific time points (*ti*). From these individual values, an average *RF*_GBP/GBPL_ is established and used in the calculation of *K*_d_. [Color figure can be viewed at wileyonlinelibrary.com]

The SLOw Mixing mOde (SLOMO)‐nMS method overcomes the problem of nonuniform RFs in nMS binding measurements performed using nanoESI (Bui et al. 2022b, 2024). The technique relies on the slow mixing of two solutions (referred to as Solutions 1 and 2) with different concentrations of the interacting species, loaded in a layered fashion into a nanoESI emitter, to establish the magnitude of both *RF* and *R*_app_ in the same experiment (Bui et al. 2022b). While the factors that control analyte mixing are not fully established, diffusion is believed to be the major driving force under the electric field strengths normally used for nanoESI (Bui et al. 2023). The assay enables the determination of *K*_d_ for both hetero‐ and homocomplexes. To apply SLOMO‐nMS to heterocomplexes, such as GBP binding to a glycoprotein ligand (*L*), two solutions with identical GBP concentrations but different ligand concentrations are loaded into the nanoESI tip (Figure 4). Before the onset of mixing, the value of *R*_*app*_ measured will reflect the equilibrium concentration ratio (*R*), established in Solution 1, and the relative *RF*. Due to the concentration gradient, the ligand concentration at the end of the tip gradually vary with time (until the solutions are fully mixed), leading to time‐dependent changes in the concentrations of GBP and GBPL, which is reflected in the changes in their measured abundances.

According to mass conservation, for the interaction shown in Equation (1a), the total concentration of GBP is constant at any time point (*ti*) (Equation 10a). Hence, the changes in the concentrations of GBP and GBPL are related, Equation (10b):

(10a) $${\left[\text{GBP}\right]}_{ti}+{\left[\text{GBPL}\right]}_{ti}={\left[\text{GBP}\right]}_{0}$$

(10b) $${\left[\text{GBP}\right]}_{t1}-{\left[\text{GPB}\right]}_{t2}={\left[\text{GBPL}\right]}_{t2}-{\left[\text{GBPL}\right]}_{t1}$$

As a result, the relative *RF* (Equation 11a) can be calculated from the ratio of the *Ab* changes (Equation 11b):

(11a) $${\mathrm{RF}}_{\text{GBP}/\text{GBPL}}={\mathrm{RF}}_{\text{GBP}}/{\mathrm{RF}}_{\text{GBPL}}$$

(11b) $${\mathrm{RF}}_{\text{GBP}/\text{GBPL}}=\Delta \mathrm{Ab}\left(\text{GBP}\right)/\Delta \mathrm{Ab}\left(\text{GBPL}\right)$$

In principle, Δ*Ab*(GBP) and Δ*Ab*(GBPL) can be measured over any time interval to calculate *RF*_GBP/GBPL_. In practice, to minimize error, multiple time points should be used to obtain an average value (Bui et al. 2022b). Once *RF*_GBP/GBPL_ is known, the concentration ratio *R* can be calculated from *R*_app_:

(12) $$R={\mathrm{RF}}_{\text{GBP}/\text{GBPL}}R_{\mathrm{app}}$$

and *K*_d_ can be found (Equations 1c) or (2a).

For homocomplexes (e.g., monomer—homodimer equilibrium, Equation 13), a dissimilar pH in Solutions 1 and 2 can be used to produce changes in *Ab*, while keeping the total GBP concentration constant.

(13) $${\text{GBPL}}_{2}⇌2\text{GBP}$$

The equilibrium shift produced from a pH change enables ${\mathrm{RF}}_{\text{GBP}/{\text{GBP}}_{2}}$, the relative *RF* of monomer (GBP) and dimer (GBP_2_), to be calculated using the following equation:

(14) $${\mathrm{RF}}_{\text{GBP}/{\text{GBP}}_{2}}=\Delta Ab\left(\text{GBP}\right)/2\Delta Ab\left({\text{GBP}}_{2}\right)$$

Once $RF_{{\text{GBP}/\text{GBP}}_{2}}$ is known, the concentration ratio (*R*) can be determined (Equation 15b) and *K*_d_ found at a single concentration (Equation 15a) or using a titration approach (Equation 15c):

(15a) $$K_{d}=\frac{{\left[\text{GBP}\right]}^{2}}{{[\text{GBP}}_{2}]}=\frac{{\left[\text{GBP}\right]}_{0}}{R\left(1+2R\right)}$$

(15b) $$R_{\mathrm{app}}=\frac{\mathrm{Ab}({\text{GBP}}_{2})}{\mathrm{Ab}(\text{GBP})}=RF_{\mathrm{GBP}/{\mathrm{GBP}}_{2}}\frac{\left[{\text{GBP}}_{2}\right]}{\left[\text{GBP}\right]}=RF_{\mathrm{GBP}/{\mathrm{GBP}}_{2}}R$$

(15c) $$\frac{2R}{2R+1}=\frac{-K_{d}-\sqrt{{K_{d}}^{2}+8{\left[\mathrm{GBP}\right]}_{0}K_{d}}}{K_{d}+\sqrt{{K_{d}}^{2}+8{\left[\mathrm{GBP}\right]}_{0}K_{d}}}$$

The effectiveness of SLOMO‐nMS has been demonstrated for a range of biomolecular interactions, including small peptide‐drug, protease‐protein inhibitor, protein oligomerization, human lectin‐nanobody, human lectin‐viral protein, bacterial lectin‐glycolipid on model membranes (Bui, Kitova et al. 2022a; Bui et al. 2022b, 2023, 2024), and bacterial membrane lectins (Gheorghita et al. 2022). One example highlighting the utility of the SLOMO‐nMS assay involved the interaction of human galectin‐7 (GAL‐7) with a galectin‐specific nanobody, which was designed to modulate immune response. GAL‐7 exists as a dimer at low micromolar concentrations, and SLOMO‐nMS enabled the GAL‐7 dimerization affinity, in the presence and absence of a glycan ligand, to be quantified. Without *RF* correction, binding measured by nMS was a factor of 70 to 180 stronger (smaller *K*_d_) than the value obtained following *RF* correction (Bui et al. 2022b).

**Figure 5:**
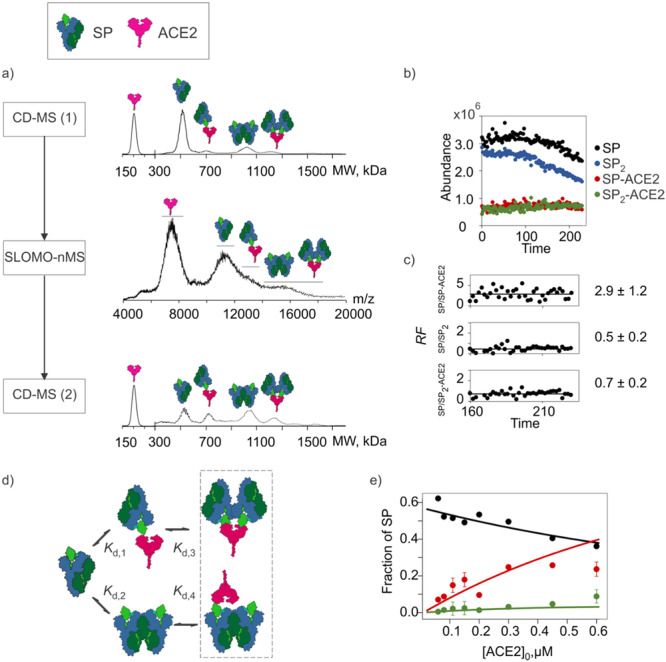
Application of SLOMO‐nMS, combined with Charge Detection MS, to quantify interactions in ammonium acetate solution (200 mM, pH 7.4) of heavily glycosylated SARS‐CoV‐2 trimeric spike protein (SP) and dimeric ACE2. (a) Workflow combining CD‐MS and SLOMO‐nMS with corresponding mass spectra. (b) The time‐resolved abundance of SP and complexes with ACE2 obtained using SLOMO‐nMS. (c) Relative *RF* (to SP) for SP‐ACE2, SP_2_, and SP_2_‐ACE2. (d) Proposed scheme of coupled equilibria and corresponding affinities. (e) Corrected (for *RF*) fractional abundance of SP, SP‐ACE2, and SP_2_‐ACE2 plotted as a function of total ACE2 concentration. Solid curves are the theoretical values calculated from the experimentally determined *K*_*d*_. Figure adapted from Bui et al. 2024. [Color figure can be viewed at wileyonlinelibrary.com]

Recently, the use of SLOMO‐nMS to quantify coupled equilibria was demonstrated (Bui et al. 2024). One of the systems studied involved the trimeric SARS‐CoV‐2 spike protein (SP) and a dimeric form of the human angiotensin‐converting enzyme 2 (ACE2), which is the main host receptor of SARS‐CoV‐2 (Avdonin et al. 2023; Clausen et al. 2020; Lan et al. 2020; Nguyen et al. 2022; Yang et al. 2020). The affinity of ACE2 for the receptor binding domain (RBD) of SP (Wuhan strain) was quantified using ITC to be 144 nM at pH 7.4 (Rombel‐Bryzek et al. 2023). Both SP and ACE2 are heavily glycosylated, with SP having 66 *N*‐ and 6 *O*‐glycosylation sites and ACE2 having 14 *N*‐ and 4 *O*‐glycosylation sites (Shajahan et al. 2023; Wang et al. 2021; Xie and Butler 2023). The heterogeneity arising from glycosylation hinders a conclusive determination, from the mass spectrum, of the interactions present in solution. To enable the identification and quantification of equilibria involving the SP and ACE2, a workflow combining charge detection (CD) using Direct Mass Technology and SLOMO‐nMS was developed (Figure 5). In addition to the SP‐ACE2 complex, signal corresponding to SP_2_ and SP_2_‐ACE2 was also detected. The SP‐ACE2 interaction (*K*_d,1_) was found to be 0.5 ± 0.2 µM while (*K*_d,2_) of SP_2_ formation was 0.3 ± 0.1 µM (Figure 5).

These and other examples highlight the broad potential of SLOMO‐nMS as a versatile tool for measuring the pathways and affinities of diverse biomolecular interactions. In the context of GBPs, SLOMO‐nMS shows particular promise for accurately quantifying interactions with glycoproteins and other glycoconjugates. This capability is especially valuable for elucidating how glycan recognition is modulated by the underlying structure. By enabling such insights, SLOMO‐nMS can significantly advance our understanding of glycan‐mediated biological processes.

#### 2.2.3 In‐Source Dissociation

Collisional heating of the gaseous ions during introduction to the MS instrument can lead to the dissociation of complexes that formed in the solution. This process, commonly referred to as in‐source dissociation, can artificially reduce the relative abundance of the GBP‐glycan complex ions and, consequently, lead to artificially high *K*_d_ values (Báez Bolivar et al. 2021; Clark and Konermann 2004; Sun et al. 2007; van Dongen and Heck 2000). In the extreme case, in‐source dissociation will produce false negatives, wherein no L‐bound GBP ions are detected. The gas‐phase stabilities of biomolecular complexes generally do not reflect solution affinities (Kitova et al. 2002b; Wang et al. 2005b; Bagal et al. 2009). The extent of in‐source dissociation is controlled by the gas‐phase stability of the complex, which is influenced, at least to some extent, by the nature of the specific interactions in solution, as well as the configuration of the mass spectrometer and the instrumental parameters used (Bagal et al. 2009; El‐Hawiet et al. 2010; Robinson et al. 1996; Sun et al. 2007; Wang et al. 2003; Xie et al. 2006). Complexes stabilized predominantly by non‐polar interactions in solution typically exhibit low kinetic stability in the gas phase. In contrast, GBP–glycan complexes are predominantly stabilized by H‐bonds, which are strengthened upon loss of hydration and confer substantial kinetic stability (Kitova et al. 2008, 2002a, 2002b). As a result, in‐source dissociation is usually only a significant problem when analyzing GBP interactions involving small (mono‐ and disaccharide) glycan ligands (Wang et al. 2005b).

**Figure 6:**
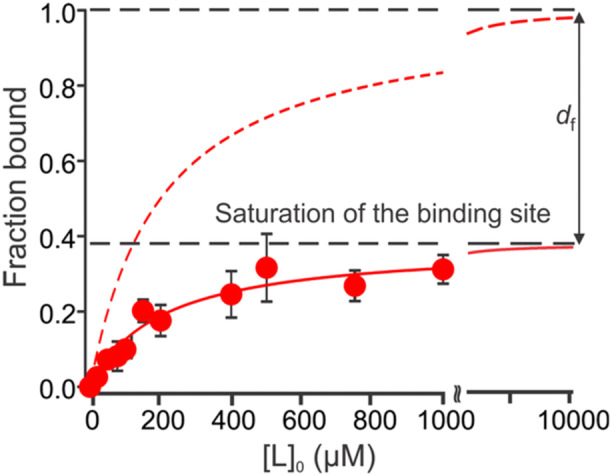
Plot of fraction of ligand (lactose)‐bound GAL‐3C versus initial ligand concentration measured by nMS. The red solid circles are the fractional abundances determined by nMS and the red solid curve is the best fit of Equation (2a) to these data. The red dashed curve shows the expected values calculated using the *K*_d_ obtained from the best fit of Equation (16). The difference between the expected and measured fraction bound values (denoted by dashed black lines) represents the magnitude of *d*_*f*_ (fraction of complex undergoing in‐source dissociation). Figure adapted from Báez Bolivar et al. 2021. [Color figure can be viewed at wileyonlinelibrary.com]

The occurrence of in‐source dissociation can usually be identified by monitoring changes in *R* arising from changes in ion source parameters that affect the internal energy of the gaseous ions. The extent of in‐source dissociation can be reduced by performing the measurements under conditions that minimize the kinetic energy of the ions during their sampling into the mass spectrometer (Kitova et al. 2012; Chernushevich and Thomson 2004). For some complexes, the choice of polarity (positive vs. negative ion mode) can significantly impact the extent of in‐source dissociation. In some instances, the use of solution and gas‐phase additives has been shown to be beneficial. For example, the addition of imidazole at concentrations > 10 mM or the introduction of imidazole vapor to the source can stabilize complexes via an evaporative cooling mechanism (Bagal et al. 2009; Liu et al. 2011; Sun et al. 2007). By performing the nMS measurements using the titration format, it may be possible to quantify the fraction of the complex ions dissociating in the ion source and, thereby, correct for this effect by introducing a dissociated fraction (*d*_*f*_) term in the fitting equation (Equation 16, Figure 6) (Báez Bolivar et al. 2021). However, this approach requires near saturation of the binding site. For low‐affinity interactions, a high concentration of a ligand is required to achieve this, which, in turn, may lead to extensive nonspecific complex formation (Báez Bolivar et al. 2021). In such cases, submicron nanoESI emitters should be used (see Section 2.2.4).

(16) $$\frac{R}{R+1}=\frac{{\left[\mathrm{GBP}\right]}_{0}+{\left[L\right]}_{0}+K_{d}-\sqrt{{\left(K_{d}-{\left[L\right]}_{0}+{\left[\mathrm{GBP}\right]}_{0}\right)}^{2}+4K_{d}{\left[L\right]}_{0}}}{2{\left[\mathrm{GBP}\right]}_{0}}(1-d_{f})$$

#### 2.2.4 Nonspecific Binding

Nonspecific binding (i.e., the formation of artificial complexes in the ESI droplets due to concentration effects) can lead to false positives (i.e., detection of complexes that do not originate from bulk solution). The presence of signal corresponding to nonspecific complexes obscures the true binding stoichiometry in solution and introduces error to the measured *K*_d_. Nonspecific complex formation can be rationalized through the charge residue model (CRM) (Dole et al. 1968). As droplets evaporate to dryness, noninteracting analytes may associate and, depending on the kinetic stability of the resulting complex, survive until MS detection. The probability of nonspecific complex formation increases with increasing analyte concentration and poses a significant challenge to quantifying weak (*K*_d_ > 100 μM) GBP–glycan interactions, as high concentration of glycan is required to produce detectable complex signal in nMS (Wang et al. 2005a; Sun et al. 2006; Shimon et al. 2010). In addition, some GBPs, such as C‐type lectins, require metal ions at high concentrations to bind glycans. High concentrations of nonvolatile salts can produce adducts that broaden the distribution of the GBP ions and suppress ion signals (Hernández and Robinson 2007). A variety of approaches have been developed to minimize nonspecific ligand binding (submicron tips [Báez Bolivar et al. 2021]) or to correct for it by using the reference protein method (Sun et al. 2006, 2007, 2009, 2010; Kitov, Han et al. 2019), and mathematical models (Kitov, Han et al. 2019; Shimon et al. 2010; Sun et al. 2006, 2007, 2009, 2010). These are briefly described below.

The most extensively used method to correct for nonspecific binding in nMS is the reference protein method, which uses a reference protein (P_ref_) that does not form specific complexes with the ligand(s) of interest (Sun et al. 2006). The technique relies on assumptions that nonspecific binding during ESI is a random process, independent of protein structure and size, and that the distribution of ligand molecules bound to P_ref_ is identical to the distribution of ligands interacting nonspecifically with the GBP (or other proteins) of interest (Sun et al. 2006). The “true” abundance of the ligand‐bound GBP (i.e., *Ab*(GBPL_*i*_)) can be calculated from the total‐apparent abundance (*Ab*_*app*_(GBPL_*i*_)) and the distribution of the bound forms of the reference protein (P_ref_L_*q*_), Equation (17):

(17) $$\mathrm{Ab}({\text{GBPL}}_{i})=(\mathrm{Ab}{\left({\text{GBPL}}_{i}\right)}_{\text{app}}-f_{1}\mathrm{Ab}({\text{GBPL}}_{i-1})-f_{2}\mathrm{Ab}({\text{GBPL}}_{i-2})-\text{…}\;-f_{q}\mathrm{Ab}({\text{GBPL}}_{i-q}))/f_{0}$$

where the fractional abundance of *q* molecules of L bound nonspecifically to P_ref_ (*f*_*q*_, where *q* ≥ 0) is calculated using the following equation:

(18) $$f_{q}=\frac{\mathrm{Ab}(P_{\text{ref}}L_{q})}{\sum_{q}\mathrm{Ab}(P_{\text{ref}}L_{q})}$$

For example, in the case of a GBP with a single ligand binding site (Equation 1a), only ion signal corresponding to GBP and GBPL species is expected from nMS analysis of a solution of GBP and L. However, nonspecific binding may lead to the detection of GBP species with more than a single bound L. The addition of a suitable P_ref_ to solution serves to both identify the occurrence of nonspecific ligand binding and to correct for the contribution of nonspecific binding to the GBP and GBPL signal. The detection of signal corresponding to L‐bound P_ref_ ions establishes that nonspecific binding of L to GBP is contributing to the mass spectrum and reveals the maximum number of L bound nonspecifically to the GBP. If P_ref_ is observed to bind one L, then GBP and GBPL are also expected to bind nonspecifically to one L. To correct for this contribution, the fractional abundances of P_ref_ (*f*_0_) and P_ref_L (*f*_1_) in the mass spectrum can be found from the measured abundances (*Ab*(P_ref_) and *Ab*(P_ref_L)), Equations (19a) and (19b):

(19a) $$f_{0}=\frac{\mathrm{Ab}(P_{\text{ref}})}{\mathrm{Ab}(P_{\text{ref}})+\mathrm{Ab}(P_{\text{ref}}L)}$$

(19b) $$f_{1}=\frac{\mathrm{Ab}(P_{\text{ref}}L)}{\mathrm{Ab}(P_{\text{ref}})+\mathrm{Ab}(P_{\text{ref}}L)}$$

The abundances of GBP, GBPL, and GBPL_2_, corrected for nonspecific binding *Ab*(GBP), *Ab*(GBPL), and *Ab*(GBPL_2_), can be found from Equation (20a):

(20a) $$\mathrm{Ab}(\text{GBP})=\mathrm{Ab}{\left(\text{GBP}\right)}_{\text{app}}/f_{0}$$

(20b) $$\mathrm{Ab}(\text{GBPL})=(\mathrm{Ab}{\left(\text{GBPL}\right)}_{\text{app}}-f_{1}\mathrm{Ab}(\text{GBP}))/f_{0}$$

(20c) $$\mathrm{Ab}({\text{GBPL}}_{2})=(\mathrm{Ab}{\left({\text{GBPL}}_{2}\right)}_{\mathrm{app}}-f_{1}\mathrm{Ab}(\text{GBPL}))/f_{0}$$

where *Ab*(GBP)_app_, *Ab*(GBPL)_app_, and *Ab*(GBPL_2_)_app_ are apparent abundances of GBP, GBPL, and GBPL_2_ in the mass spectrum. Notably, for a GBP with a single glycan binding site, *Ab*(GBPL_2_) is expected to be near zero after correction for nonspecific binding.

**Figure 7:**
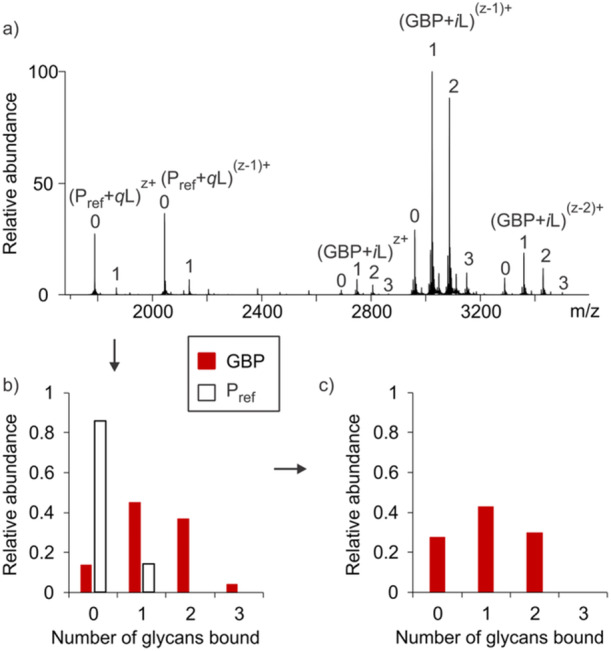
Illustration of the implementation of the reference protein method for correcting ESI mass spectra for the occurrence of nonspecific ligand binding. (a) Illustrative nanoESI mass spectrum acquired for a solution of GBP (with 2 specific binding sites), a glycan ligand (L) and a P_ref_. The number of L bound to P_ref_ is denoted as *q* and number bound to GBP as *i*. (b) Relative abundances of (GBP + *i*L) and (P_ref_ + *q*L) determined directly from the mass spectrum and (c) after correction for nonspecific binding using the distribution of (P_ref_ + *q*L). [Color figure can be viewed at wileyonlinelibrary.com]

An illustration of the changes in the distribution of L‐bound GBP species, before and after correction for nonspecific binding using the reference protein method, is shown in Figure 7. In this instance, galectin‐1, which possesses two equivalent binding sites, served as the GBP and 3'‐sialyllactose was the ligand. At elevated ligand concentration, three bound ligands were detected by nMS. However, upon correction of the mass spectrum using the distribution of ligands bound to P_ref_ (Equations 19a and 20a), it is found that a maximum of two 3'‐sialyllactose molecules are bound in solution.

(20d) $$\mathrm{Ab}\left({\text{GBPL}}_{3}\right)=\left(\mathrm{Ab}{\left({\text{GBPL}}_{3}\right)}_{\mathrm{app}}-f_{1}\mathrm{Ab}\left({\text{GBPL}}_{2}\right)\right)/f_{0}$$

As expected, the corrected abundance of the GBPL_3_ complex was found to be zero, within experimental error, indicating that this complex does not exist in solution.

The reference protein method has been extensively tested using a variety of GBP–glycan interactions and shown to be generally valid (Sun et al. 2007, 2006, 2010, 2009). While nonspecific binding is independent of the protein's size and structure, the occurrence of in‐source dissociation, which may lead to nonuniform losses of nonspecific ligands from the GBP and P_ref_, can introduce error to the correction (Han et al. 2018). Consequently, it is a best practice to use a P_ref_ that has properties (in particular MW) similar to those of the GBP of interest.

The reference protein method has been used in the study a number of low‐affinity GBP‐glycan interactions. For example, the weak interactions (with *K*_d_ between 2.5 mM and 300 µM) between the recombinant P dimer of human noroviruses (NoVs) and the histo‐blood group antigens (HBGAs) were precisely quantified (Han et al. 2013), as were the affinities of a library of the 20 most abundant HMOs for a series of bacterial exotoxins (Shiga toxin type 2 holotoxin and the B subunit homopentamers of cholera toxin, heat‐labile toxin and Shiga toxin type 1) (El‐Hawiet et al. 2015).

An alternative approach for correcting mass spectra for the occurrence of nonspecific ligand binding, introduced by Sharon and colleagues, employs a mathematical model (Shimon et al. 2010). The method, which treats nonspecific binding as independent interactions with a common binding constant (*K*_*n*_), can be applied in cases where the number of binding sites for the target protein is known in advance. Application of this method in an nMS study of the stepwise binding of HBGAs (B trisaccharide and B type 1 tetrasaccharide) to recombinant P dimer of human NoVs produced nonspecific binding corrections similar to those obtained with the reference protein method (Han et al. 2018). Using these two independent approaches, it was definitively shown by nMS (Han et al. 2018) that a cooperative HBGA binding model, previously suggested based on findings obtained with STD‐NMR spectroscopy, was incorrect (Mallagaray et al. 2015).

As opposed to using after‐the‐fact correction‐based approaches, the problem of nonspecific ligand binding in affinity measurements can be minimized by reducing the size of the initial droplets formed in the (nano)ESI processes (Wang et al. 2005a). The size of the droplets produced depends mainly on solution flow rate, which is controlled by several factors, such as the size of the emitter orifice and the spray voltage and current (Davidson et al. 2017; Hollerbach et al. 2018; Schmidt et al. 2003; Xia and Williams 2019). The typical nanoESI emitters used to perform GBP–glycan binding measurements produce solution flow rates in the nL min^−1^ range (Schmidt et al. 2003). Since the flow rate was shown to be proportional to the emitter i.d (Li and Cole 2003; Mortensen and Williams 2016) and the initial droplets have the size of 5%–10% of the orifice i.d (Xia and Williams 2019), the initial droplets produced with the typical nanoESI emitters have diameters in the range of tens to hundreds of nm (Schmidt et al. 2003; Wang et al. 2003b).

**Figure 8:**
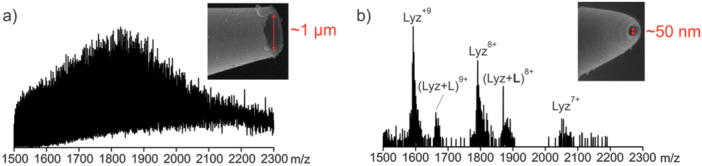
Representative nanoESI mass spectra acquired in positive ion mode for a solution of lysozyme (Lyz, 10 µM) and *N*‐acetylglucosamine tetrasaccharide (L, 20 µM) in 1X PBS (137 mM NaCl, 2.7 mM KCl, 10 mM Na_2_HPO_4_ and 1.8 mM KH_2_PO_4_) using a (a) standard nanoESI emitter (i.d. ~1 μm) and a (b) submicron emitter (i.d. ~50 nm). Figures adapted from Báez Bolivar et al. 2021. [Color figure can be viewed at wileyonlinelibrary.com]

Several groups have successfully produced submicron emitters, which have i.d. < μm (Agasid et al. 2021; Báez Bolivar et al. 2021; Nguyen et al. 2019; Panczyk et al. 2020; Susa et al. 2018, 2017a, 2017b; Yuill et al. 2013). These emitters significantly reduce nonspecific binding and allow affinity measurements in native‐like buffer with high concentrations of nonvolatile salts (Nguyen et al. 2019; Susa et al. 2018, 2017a, 2017b; Yuill et al. 2013) (Figure 8). Moreover, submicron emitters enable the use of high glycan ligand concentrations, up to several mM, which is critical for measuring very weak GBP–glycan interactions (Jung et al. 2021; Schmidt et al. 2024). These high concentrations can be analyzed without significant nonspecific binding, ensuring accurate and reliable measurements of these otherwise challenging interactions (Báez Bolivar et al. 2021).

### 2.3 Indirect Quantification of GBP–Glycan Interactions. Competitive nMS Assays

**Figure 9:**
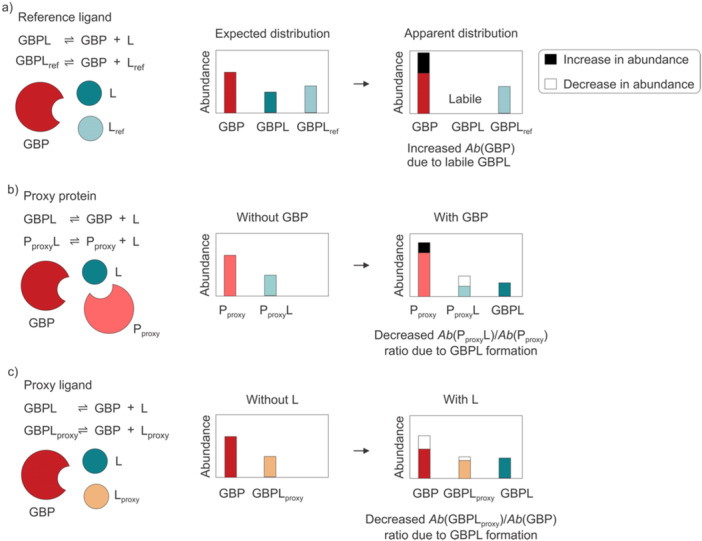
Overview of the quantitative nMS methods employing competitive binding described in this review: (a) reference ligand method, (b) proxy protein method, and (c) proxy ligand method. [Color figure can be viewed at wileyonlinelibrary.com]

The nMS assay relies on the direct detection and quantification of both free and ligand‐bound GBP ions. However, its application to kinetically labile complexes, which undergo dissociation in the source, can be challenging. Additionally, accurate determination of the relative abundance of bound versus free GBP may not be possible in the case of high MW or heterogeneous GBPs, due to signal overlap of GBP and GBPL complex ions. In such situations, nMS can be combined with competitive GBP–glycan binding strategies to quantify *K*_d_ (El‐Hawiet, Kitova et al. 2012, 2010; Han, Kitova, Li et al. 2015; Liu, Bai et al. 2012). Three different approaches have been developed: the reference ligand method, the proxy protein method and the proxy ligand method. These methods are illustrated in Figure 9 and described below.

#### 2.3.1 Reference Ligand Method

The reference ligand method was developed to quantify GBP–glycan complexes that are susceptible to in‐source dissociation (El‐Hawiet et al. 2010). This approach uses a reference ligand (L_ref_), which binds specifically to the same binding site on the GBP as the ligand of interest (L), with a known *K*_d_ and forms a stable complex in the gas phase. Monitoring the binding of GBP to L_ref_ enables accurate quantification of L binding.

In the absence of in‐source dissociation, the affinities of the GBPL and GBPL_ref_ complexes can be expressed using the following equations:

(21a) $$K_{d}=\frac{{\left[L\right]}_{0}-\frac{R}{1+R+R_{\text{ref}}}{\left[\mathrm{GBP}\right]}_{0}}{R}$$

(21b) $$K_{d,\text{ref}}=\frac{{\left[L_{\text{ref}}\right]}_{0}-\frac{R_{\text{ref}}}{1+R+R_{\text{ref}}}{\left[\mathrm{GBP}\right]}_{0}}{R_{\text{ref}}}$$

where *R* is given by Equation (2) and *R*_ref_ is given by the following equation:

(22) $$R_{\mathrm{ref}}=\frac{\left[{\mathrm{GBPL}}_{\text{ref}}\right]}{\left[\mathrm{GBP}\right]}$$

If the GBPL complex undergoes in‐source dissociation, *R* no longer reflects the *Ab* ratio of GBPL‐to‐GBP concentration in solution as the *Ab* of free GBP increases due to loss of L. The apparent *Ab* ratio of GBPL_ref_ and GBP (*R*_ref,app_) can be expressed by the following equation:

(23) $$R_{\mathrm{ref},\mathrm{app}}=\frac{\left[{\mathrm{GBPL}}_{\text{ref}}\right]}{[\mathrm{GBP}]+{\left[\mathrm{GBPL}\right]}_{\mathrm{dissociated}}}=\frac{R_{\text{ref}}}{1+R}$$

where [GBPL]_dissociated_ is the equivalent GBPL concentration that corresponds to the amount of GBPL that undergoes dissociation in the gas phase. In cases where the GBPL is completely dissociated in the gas phase (no GBPL ion signal is detected) [GBPL]_dissociated_ is the concentration of GBPL at equilibrium. As *K*_d,ref_ is known, the “true” *R*_ref_ can be obtained from the following equation:

(24) $$R_{\text{ref}}=\frac{{\left[L_{\text{ref}}\right]}_{0}-\frac{R_{\text{ref},\text{app}}}{R_{\text{ref},\text{app}}+1}{\left[\mathrm{GBP}\right]}_{0}}{K_{d,\text{ref}}}$$

and *R* can be obtained by rearranging Equation (23) to the form shown in the following equation:

(25) $$R=\frac{R_{\text{ref}}}{R_{\text{ref},\text{app}}}-1$$

In cases where GBPL is only partially dissociated in the source, *R*_ref_ can be calculated from Equation (26), where *R*_app_ is given by Equation (27):

(26) $$R_{\text{ref}}=\frac{{\left[L_{\text{ref}}\right]}_{0}-\frac{R_{\text{ref},\text{app}}}{R_{\text{ref},\text{app}}+R_{\text{app}}+1}{\left[\mathrm{GBP}\right]}_{0}}{K_{d,\text{ref}}}$$

(27) $$R_{\text{app}}=\frac{\mathrm{Ab}(\mathrm{GBPL})}{\mathrm{Ab}(\mathrm{GBP})}=\frac{[\mathrm{GBPL}]-{\left[\mathrm{GBPL}\right]}_{\mathrm{dissociated}}}{\left[\mathrm{GBP}\right]}$$

The true *R* is then calculated from *R*_ref,app_ and *R*_ref_, Equation (28):

(28) $$R=\frac{\left(1+R_{\text{app}}\right)}{R_{\text{ref},\text{app}}}R_{\text{ref}}-1$$

The reference ligand method can also be used in cases where the GBP has multiple binding sites. For example, when there are two binding sitse, the corresponding *K*_d_ (*K*_d,1_ and *K*_d,2_) can be calculated from the following equations:

(29a) $$K_{d,1}=\frac{{\left[L\right]}_{0}-\frac{R_{1,0}+2R_{2,0}+R_{1,1}}{1+R_{1,0}+R_{2,0}+R_{0,1}+R_{1,1}+R_{0,2}}{\left[\mathrm{GBP}\right]}_{0}}{R_{1,0}}$$

(29b) $$K_{d,2}=R_{1,0}\left(\frac{{\left[L\right]}_{0}-\frac{R_{1,0}+2R_{2,0}+R_{1,1}}{1+R_{1,0}+R_{2,0}+R_{0,1}+R_{1,1}+R_{0,2}}{\left[\mathrm{GBP}\right]}_{0}}{R_{2,0}}\right)$$

where *R*_*i*,*j*_ is the relative *Ab* ratio of GBP bound to *i* and *j* molecules of L and L_ref_, respectively, to free GBP, Equation (30):

(30) $$R_{i,j}=\frac{\left[{\mathrm{GBPL}}_{i}L_{\mathrm{ref},j}\right]}{\left[\mathrm{GBP}\right]}$$

#### 2.3.2 Proxy Protein Method

The proxy protein method was developed to quantify ligand interactions involving high MW proteins and protein complexes that are not readily detected by nMS (El‐Hawiet, Kitova et al. 2012). It relies on a proxy protein (P_proxy_), which binds to the ligand of interest (L) with known affinity and for which the P_proxy_ L complex can be directly detected and quantified by nMS. The addition of the GBP of interest to the solution of P_proxy_ and L will result in reduction of the measured *Ab* ratio (*R*_proxy_) of ligand‐bound to free P_proxy_ ions (i.e., [P_proxy_L]/[P_proxy_]). The affinity of L for the GBP can be calculated from the measured value of *R*_proxy_ and the equation of mass balance, Equation (31):

(31) $${\left[L\right]}_{0}=\left[L\right]+\left[P_{\mathrm{proxy}}L\right]+\sum_{1\leq i\leq q}i\left[{\mathrm{GBPL}}_{i}\right]$$

where GBP has *q* binding sites and $\sum_{1\leq i\leq q}i[{\mathrm{GBPL}}_{i}]$ is the total concentration of L bound to GBP.

The concentration of free L can be calculated from (*R*_proxy_) and *K*_d,proxy_, Equation (32):

(32) $$[L]=K_{d,\text{proxy}}R_{\text{proxy}}$$

while the concentration of P_proxy_L can be found from the following equation:

(33) $$[P_{\mathrm{proxy}}L]=\frac{{\left[P_{\mathrm{proxy}}\right]}_{0}R_{\mathrm{proxy}}}{R_{\mathrm{proxy}}+1}$$

By substituting Equations (32) and (33) into Equation (31), $\sum_{1\leq i\leq q}i[{\mathrm{GBPL}}_{i}]$ can be calculated, Equation (34):

(34) $$\sum_{1\leq i\leq q}i\left[{\mathrm{GBPL}}_{i}\right]={\left[L\right]}_{0}-\frac{\left[P_{\mathrm{proxy}}\right]R_{\mathrm{proxy}}}{R_{\mathrm{proxy}}+1}-K_{d,\mathrm{proxy}}R_{\mathrm{proxy}}$$

In general, the *K*_d_ for the GBPL complex can be described by the following equation:

(35) $$K_{d}=\frac{{(q\left[\text{GBP}\right]}_{0}-\sum_{1\leq i\leq q}i\left[{\mathrm{GBPL}}_{i}\right])\left[L\right]}{\sum_{1\leq i\leq q}i\left[{\mathrm{GBPL}}_{i}\right]}$$

where [L] and $\sum_{1\leq i\leq q}i[{\mathrm{GBPL}}_{i}]$ are obtained from Equations (32) and (34), respectively; *K*_d_ can then calculated from the following equation:

(36) $$K_{d}=\frac{{(q\left[\text{GBP}\right]}_{0}-{\left[L\right]}_{0}+\frac{\left[P_{\mathrm{proxy}}\right]R_{\mathrm{proxy}}}{R_{\mathrm{proxy}}+1}+K_{d,\mathrm{proxy}}R_{\mathrm{proxy}})K_{d,\mathrm{proxy}}R_{\mathrm{proxy}}}{{\left[L\right]}_{0}-\frac{\left[P_{\mathrm{proxy}}\right]R_{\mathrm{proxy}}}{R_{\mathrm{proxy}}+1}-K_{d,\mathrm{proxy}}R_{\mathrm{proxy}}}$$

In cases where *q* is known, *K*_d_ can be estimated from a single measurement, although a titration approach is required to solve for both *K*_d_ and *q*.

The proxy protein method has been employed to quantify the affinity of HBGAs for NoV VA387 virus‐like particles (MW ~ 10 MDa) and the associated subviral P particle (24‐mer of the protruding domain of the capsid protein, MW 865 kDa) (Han, Kitova, Tan et al. 2015a). This method was also used to screen libraries of HBGAs and HMOs against an N‐terminal fragment of the family 51 carbohydrate‐binding module, a fucose‐binding lectin from *Ralstonia solanacearum*, and human NoV VA387 P particle (Han et al. 2016). Additionally, the proxy protein method forms the basis of the Competitive Universal Proxy Receptor Assay (CUPRA), a quantitative glycan library screening technique developed to establish the glycan‐binding specificity of GBPs (Kitov et al. 2019b).

**Figure 10:**
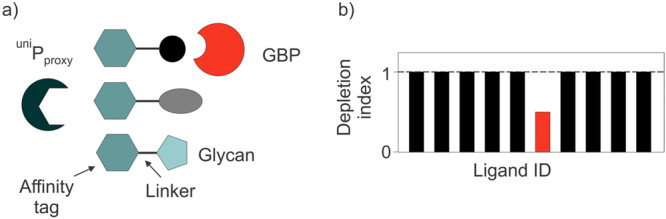
Overview of the Competitive Universal Proxy Receptor Assay (CUPRA) for quantitative screening of libraries of labeled glycans against a GBP using nMS. (a) Each ligand consists of an affinity tag, a linker and oligosaccharide. The affinity tag is the same for each ligand and is recognized by the universal proxy protein receptor (^Uni^P_proxy_). (b) The relative abundance of each ligand‐bound ^Uni^P_proxy_ species is measured in the presence and absence of the target GBP. The corresponding ratio is referred to as the depletion index (DI). When there is no interaction with GBP, DI ≈ 1.0; when there is an interaction, DI < 1.0. [Color figure can be viewed at wileyonlinelibrary.com]

The CUPRA method (Figure 10) uses a library of hetero‐bifunctional compounds, each consisting of oligosaccharides linked to a common affinity tag, and utilizes nMS to measure coupled binding equilibria involving the glycan library, a universal proxy protein receptor (^Uni^P_proxy_)—which binds all library components through the affinity tag—and the target GBP. Changes in the relative abundances of ^Uni^P_proxy_ complexes upon the introduction of a GBP to the solution (referred to as the depletion index) are used to identify ligand binding to the GBP. Importantly, direct detection of the target GBP is not required, making CUPRA applicable to any GBP, regardless of size or heterogeneity. The analysis of these changes, using a binding model that accounts for all potential interactions between the glycan library components, GBP and ^Uni^P_proxy_, provides the affinities of the detected GBP ligands.

#### 2.3.3 Proxy Ligand Method

The proxy ligand method was originally developed to quantify GBP interactions with glycolipids (GLs). As discussed in more detail below (Section 5), nMS studies of GBP–GL interactions typically involve the incorporation of the GL into a soluble model membrane, such as a nanodisc (ND), picodisc or micelle (Han et al. 2017). Upon transfer to the gas phase, GBP–GL complexes present in the solution (and associated with the model membrane) detach from the membrane due to Coulombic repulsion (Han, Kitova, Li et al. 2015). The free GBP and GBP–GL complex undergo different ionization pathways (from solution to the gas phase) and, hence, have nonuniform *RF*s. This introduces error to the affinity measurement. The proxy ligand nMS assay utilizes a proxy ligand (L_proxy_) that binds to GBP of interest with known affinity (*K*_d_,_proxy_). The interaction of the GL to the GBP can be quantified by monitoring the relative abundance of GBPL_proxy_ using direct nMS measurements. And *K*_d_ can be calculated using the following equation:

(37) $$\begin{array}{l} K_{d}=({\left[L_{\mathrm{proxy}}\right]}_{0}-K_{d,\mathrm{proxy}}R_{\mathrm{proxy}})\\ \times \left(\frac{{\left[L\right]}_{0}}{R_{\mathrm{proxy}}{\left[\mathrm{GBP}\right]}_{0}-({\left[L_{\mathrm{proxy}}\right]}_{0}-K_{d,\mathrm{proxy}}R_{\mathrm{proxy}})(R_{\mathrm{proxy}}+1)}-\frac{1}{R_{\mathrm{proxy}}}\right) \end{array}$$

where *R*_proxy_ is calculated using the following equation:

(38) $$R_{\mathrm{proxy}}=\frac{\left[{\mathrm{GBPL}}_{\mathrm{proxy}}\right]}{\left[\mathrm{GBP}\right]}$$

As described in Section 5.1, the proxy ligand method has enabled the quantification of the interactions of GBPs with GLs embedded in a variety of different model membranes (Han, Kitova, Li et al. 2015, Han et al. 2017; Han, Kitov et al. 2020).

## 3 Variable‐Temperature (VT)‐nMS to Quantify GBP–Glycan Binding Thermodynamics

Most nMS GBP–glycan binding studies are conducted at room temperature, allowing the *K*_d_ and the corresponding Gibbs free energy change (∆*G*) for the dissociation reaction to be determined at that temperature. By performing these measurements across a range of temperatures using a temperature‐controlled nanoESI device, it becomes possible to estimate the enthalpy (∆*H*) and entropy changes (∆*S*) based on the temperature dependence of *K*_d_. A variety of different temperature‐controlled nanoESI devices have been reported, and a comprehensive review of the designs can be found elsewhere (Alexander Harrison et al. 2021). Most often, the solution temperature is controlled by placing the nanoESI emitter in contact with a temperature‐controlled metal (aluminum or copper) block.

In cases where ∆*H* and ∆*S* are temperature‐independent (at least over the temperature range considered), the linear form of the van't Hoff equation can be used to describe the temperature dependence of *K*_d_, Equation (39):

(39) $$\ln\;K_{d}=\frac{\Delta H}{RT}-\frac{\Delta S}{R}$$

where **R** is the ideal gas constant. However, ∆*H* and ∆*S* often exhibit a measurable temperature dependence, and a plot of ln*K*_d_ and 1/*T* will be nonlinear. In such cases, the temperature dependence of *K*_d_ can be described by the following equation:

(40) $$\ln\;\frac{K_{d,0}}{K_{d}}=-\frac{\Delta H_{0}-T_{0}\Delta C_{p}}{R}\left(\frac{1}{T_{0}}-\frac{1}{T}\right)+\frac{\Delta C_{p}}{R}\;\ln\;\frac{T}{T_{0}}$$

where *T*_0_ is an arbitrarily chosen reference temperature and *K*_d,0_ and ∆*H*_0_ are the *K*_d_ and ∆*H* at that temperature and ∆*C*_*p*_ is temperature‐independent heat capacity change.

**Figure 11:**
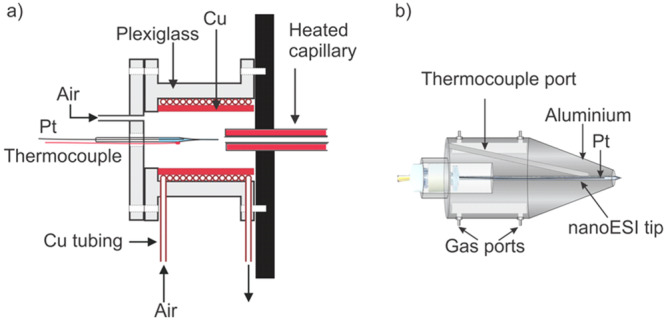
Two temperature‐controlled nanoESI devices used for GBP–glycan affinity measurements performed using VT‐nMS. (a) The nanoESI glass tip is inserted into a Plexiglas chamber lined with a copper sleeve, which is in thermal contact with a copper coil that is heated or cooled by circulating air. A portion of the temperature‐regulated air is introduced into the chamber to satisfy the gas intake requirement of the ion source. The temperature inside the chamber, which is monitored by a thermocouple in contact with the nanoESI tip, can be controlled within ±1.5°C from 5°C to 40°C. (b) The nanoESI tip is inserted into the central channel (i.d. 1.2 mm) of a tapered aluminum block. The end of the tip extends ~1 mm from the block. The desired temperature is achieved through the combined effects of cooling and heating. Cooling process is achieved by varying the flow rate of nitrogen gas that passes through copper tubing in contact with a dry ice/ethanol bath and then through two symmetric channels at the outer edges of the device. Controlled heating is achieved using a pair of cartridge heaters embedded into two symmetric channels. The temperature of the device is measured by a thermocouple introduced through the angled channel and placed in proximity of the end of the nanoESI tip. Figures adapted from Daneshfar et al. 2004 and Kitov et al. 2019b. [Color figure can be viewed at wileyonlinelibrary.com]

**Figure 12:**
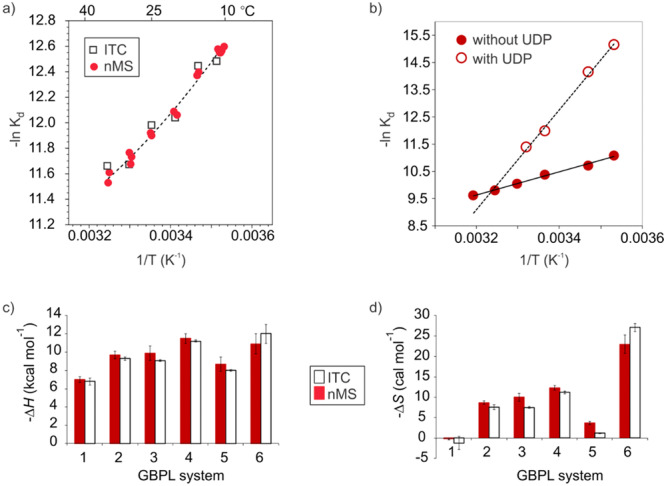
Illustration of (a) nonlinear (Equation 40) and (b) linear (Equation 39) van't Hoff equation fitting to nMS‐derived *K*_d_ measured for the Se155‐4 scFv with Galα[Abe]Man and human blood group α‐(1→2)‐*N*‐acetylgalactosaminyltransferase with α‐l‐Fuc*p*‐(1→2)‐β‐d‐Gal*p*‐O(CH_2_)_7_CH_3_. For comparison, ITC‐derived values are also shown in (a). Dashed curves are the best fits of Equations (40) and (39) to the experimental data in (a) and (b) respectively. (c and d) Comparison of the ∆*H* and ∆*S* values (at 25°C) obtained from ITC and nMS measurements. Figures adapted from Daneshfar et al. 2004 and Shoemaker et al. 2008. [Color figure can be viewed at wileyonlinelibrary.com]

The first demonstration of VT‐nMS for quantifying the thermodynamic parameters of biomolecular interactions was reported by Klassen and coworkers (Daneshfar et al. 2004). Using a homebuilt nanoESI device employing an airflow to regulate the nanoESI emitter/solution temperature (Figure 11a,b), the thermodynamic parameters (∆*H* and ∆*S*) of a series of GBP–glycan complexes were determined by fitting Equation (40) to the temperature‐dependent *K*_d_ values determined at solution temperatures ranging from 5°C to 40°C. One of the systems investigated was a single chain variable fragment (scFv) of the monoclonal antibody Se155‐4 and its native trisaccharide ligand, Galα(1→2)[Abe α(1→3)]Man (Sigurskjold et al. 1991). Comparison of van't Hoff plots constructed from *K*_d_ measured by VT‐nMS and ITC (Figure 12a) showed excellent agreement (Daneshfar et al. 2004; Sigurskjold et al. 1991). Moreover, the 25°C ∆*H* values determined with VT‐nMS and ITC agreed within 10% (Figure 12c,d). In a subsequent study, the device was used to probe the temperature‐dependent cooperativity of acceptor substrate (α‐L‐Fuc*p*‐(1→2)‐β‐D‐Gal*p*‐O(CH_2_)_7_CH_3_) binding to the human blood group glycosyltransferases in the presence and absence of uridine 5'‐diphosphate (UDP) and Mn^2+^ (Shoemaker et al. 2008). The corresponding ∆*H* and ∆*S* values were determined using linear van't Hoff equation fitting (Figure 12b). It was found that adding UDP and Mn^2+^ reduced ∆*H* and ∆*S* values significantly but, interestingly, ∆*G* was unaffected at physiological temperature (37°C).

In addition to allowing the determination of thermodynamic parameters of GBP–glycan binding, VT‐nMS can also aid in the detection of weak interactions. As ∆*H* for most GBP–glycan complexes is exothermic (negative), reducing the solution temperature increases the concentration of complex, thereby facilitating detection. For example, CUPRA‐based screening performed at 0° C facilitated the detection of ligands that were difficult or impossible to detect at room temperature (Kitov et al. 2019b). Similarly, CaR‐nMS screening of natural libraries of *N*‐glycans carried out at low temperatures has been shown to greatly increase the number of ligands detected (Park et al. 2020).

## 4 nMS‐Based High Throughput Glycan Library Screening

The ability to monitor multiple interactions simultaneously makes nMS well‐suited for high‐throughput screening of glycan libraries against GBPs. There are two general approaches to nMS screening—direct and indirect ligand detection. In the direct approach, ligands are identified by detecting GBP–glycan complexes produced from a solution containing both the GBP and the glycan library (Cederkvist et al. 2006; Cheng et al. 1995; El‐Hawiet, Shoemaker et al. 2012; Gao et al. 1996). This approach is ideal for small GBPs and defined libraries consisting of MW‐unique components. For large or heterogeneous GBPs (e.g., virus particles and glycosylated GBPs), resolving the peaks of complexes and free GBP can be challenging, even with high resolution mass analyzers. Such peak overlap complicates both the identification and quantification of GBP–glycan interactions. To address this challenge, the CaR‐nMS assay was developed (El‐Hawiet, Shoemaker et al. 2012).

### 4.1 CaR‐nMS Glycan Library Screening

**Figure 13:**
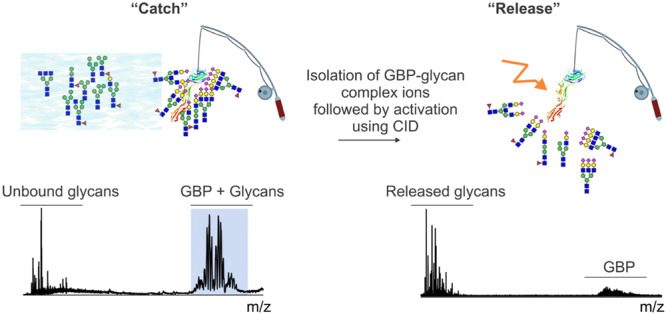
Cartoon representation of the Catch‐and‐Release (CaR)‐nMS assay. In CaR‐nMS, the target GBP is used as the bait to “catch” any glycan ligands present in the mixture. All components of the mixture are transferred to the gas phase using (nano)ESI. Ions corresponding to GBP‐glycan complexes are then isolated (from ions of unbound glycans) and dissociated using CID. The glycan ligands released as ions are then detected. [Color figure can be viewed at wileyonlinelibrary.com]

In CaR‐nMS, the gaseous ions corresponding to GBP–glycan complexes produced from a solution of GBP and glycan library are isolated in a first stage of MS, then dissociated, usually using collision‐induced dissociation (CID), and the released glycan ligand ions detected (Figure 13). CaR‐nMS is typically performed in negative ion mode to enable glycans to efficiently compete for charge with the GBP. Since free glycans are typically small (< 5 kDa) compared to the GBP, the released ligands are detected at a low *m/z* range (approximately 100–2000). A resolving power of 25,000 is generally sufficient to distinguish structures with small (1 Da) differences in MW. Due to the nonuniform detection efficiency of free glycans and GBP ions, CaR‐nMS is a semiquantitative approach. However, when performed with a glycan library at equimolar concentrations, the intensity of released glycans can be used for affinity ranking. Applications of CaR‐nMS for glycan library screening, which have been reviewed elsewhere (Bui et al. 2022a), have led to the discovery of glycan ligands of numerous GBPs, including antigen‐binding fragments, bacterial toxins, plant lectins, human proteins, and viral proteins (El‐Hawiet et al. 2013; Nguyen et al. 2022; Park et al. 2020).

The CaR‐nMS assay was originally conceived for screening defined libraries consisting of glycans prepared predominantly by chemical or chemo‐enzymatic synthesis (El‐Hawiet et al. 2013; El‐Hawiet, Shoemaker et al. 2012; Zhang et al. 2012). However, it is ideally suited for shotgun glycomics (SG) screening, which developed to overcome the limited availability of purified glycans and utilizes natural libraries from biological sources (e.g., biofluids, cultured cells and tissue) (Song et al. 2011). The method is usually performed using glycan microarrays produced from fractionated and labeled glycans printed on a glass slide. Microarray‐based SG is a powerful screening tool and has led to the discovery of ligands of a variety of endogenous and exogenous GBPs (Heimburg‐Molinaro et al. 2024). However, microarray screening is not quantitative and has limitations, such as binding artifacts associated with GBP and glycan modifications and immobilization and false negatives for low‐affinity ligands (Grant et al. 2014; Kilcoyne et al. 2012; Smith et al. 2019). Moreover, it is difficult to generate purified glycans from natural libraries, which results in mixtures of structures on the array and can confound interpretation (Casalena et al. 2012; Grant et al. 2014; Kilcoyne et al. 2012; van Diepen et al. 2012). Implementation of the CaR‐nMS assay, in contrast, does not require fractionation, labeling or immobilization. The first demonstration of CaR‐nMS‐based SG involved screening HMOs from pooled donor breast milk against GAL‐3C and a fragment of the blood group antigen‐binding adhesion (BabA) from *Helicobacter pylori* (El‐Hawiet et al. 2018). Using ion mobility separation arrival times and CID fingerprints of released glycans, novel ligands of BabA were identified by CaR‐nMS screening.

Because the concentration of individual glycans in the natural libraries is unknown; neither absolute nor relative affinities can be directly determined by CaR‐nMS screening. In principle, affinity rankings can be obtained by normalizing the relative Ab of released glycans measured by CaR‐nMS with relative concentrations of the glycans measured by liquid chromatography (LC) analysis of the library following labeling with a fluorophore (Park et al. 2020). However, in practice, this approach is limited by nonuniform efficiency in the labeling and purification steps, which causes significant deviation in relative concentrations in the unlabeled and labeled libraries (Bui et al. 2022a). The label also influences, in a nonuniform way, the ligand release efficiency in CaR‐nMS. Moreover, the presence of a fluorophore on the glycan generally weakens their GBP interactions, again in a nonuniform manner (Bui et al. 2022a).

The uncertainties in relative affinities introduced by glycan labeling underscore the need for a label‐free method to reliably rank GBP‐glycan affinities from natural libraries using CaR‐nMS. To address this, the Concentration‐Independent (COIN) method was developed (Bui et al. 2023). The assay, a label‐free approach for quantitative shotgun glycomics (SG), builds on a serial dilution strategy implemented with SLOMO‐nMS (Bui et al. 2022b).

### 4.2 Concentration‐Independent (COIN)‐nMS for Quantitative Shotgun Glycomics

**Figure 14:**
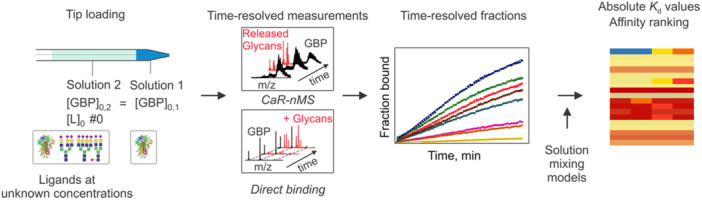
Overview of the COIN‐(CaR)‐nMS assay for quantitative SG screening. The standard workflow involves the following steps: Step (1) tip loading: two solutions (Solutions 1 and 2) are loaded to the nanoESI emitter; the target GBP is present at the same concentration in both solutions, while the glycan library of unknown concentration is only present in Solution 2. Step (2) time‐resolved nMS measurements: glycan binding detected directly (i.e., detection of glycan‐bound GBP ions) or indirectly by CaR (i.e., detection of glycans release from the GBP ions by CID). Step (3) time‐dependent fraction of GBP bound to each detected ligand: constructed from the time‐resolved nMS data. Step (4) data processing: application of a mathematical model to fit the data time‐dependent fractional binding data to obtain absolute *K*_d_ values, from which an affinity ranking is established. [Color figure can be viewed at wileyonlinelibrary.com]

To implement COIN‐nMS for quantitative screening of a natural glycan library, the target GBP is added to both Solution 1 and Solution 2 at the same concentration and the glycans, at unknown concentrations, are added to Solution 2. Mixing creates a glycan concentration gradient inside of the emitter, which leads to time‐dependent changes in glycan ligand binding (Figure 14). Different models can be used to approximate the concentration change. The simplest model assumes a linear dependence of concentration on mixing time (Bui et al. 2023). This model generally holds for a certain time interval after mixing (Bui et al. 2023). The length of the interval depends on the ligand concentration in Solution 2.

Under conditions where the change in glycan concentration is approximately linear, the concentration of glycan at a given (mixing) time point ([L]_*t*_) can be described by the following equation:

(41a) $${\left[L\right]}_{t}=b+C_{L}t$$

where *b* is the mixing point (i.e., time point when mixing starts at the end of the tip, as established from the time‐resolved binding data). When changes in the concentration are considered from the onset of mixing, *b* becomes 0 and Equation (41a) can be rewritten as Equation (41b):

(41b) $${\left[L\right]}_{t}=C_{L}t$$

when the [L]_0_ term is replaced with [L]_*t*_, Equation (2) can be rewritten as the following equation:

(42a) $$\frac{R_{t}}{R_{t}+1}=\frac{{\left[\mathrm{GBP}\right]}_{0}+C_{L}t+K_{d}-\sqrt{{\left(K_{d}-C_{L}t+{\left[\mathrm{GBP}\right]}_{0}\right)}^{2}+4K_{d}C_{L}t}}{2{\left[\mathrm{GBP}\right]}_{0}}$$

where *R*_*t*_ is the *Ab* ratio (Equation 42b) at time point *t*:

(42b) $$R_{t}=\frac{\mathrm{Ab}{\left(\text{GBPL}\right)}_{t}}{\mathrm{Ab}{\left(\text{GBP}\right)}_{t}}$$

COIN‐nMS can also be applied using CaR‐nMS. In this case, because GBPL complexes are dissociated by the loss of ligand L to produce free GBP ions, the fraction of GBP bound by glycan (*R*/*R *+ 1) is calculated as in the following equation:

(43) $$\frac{R_{t}}{R_{t}+1}=\frac{{\mathrm{Ab}}_{t}\left(L\right)}{{\mathrm{Ab}}_{t}\left(\mathrm{GBP}\right)}$$

where *Ab*_*t*_(L) and *Ab*_*t*_(GBP) are the time‐dependent *Ab* of the released glycan and GPB ions, respectively. Due to nonuniform detection efficiencies, Equation (42a) is re‐written as the following equation:

(44) $$\frac{R_{t}}{R_{t}+1}=\text{DE}\frac{{\left[\mathrm{GBP}\right]}_{0}+C_{L}t+K_{d}-\sqrt{{\left(K_{d}-C_{L}t+{\left[\mathrm{GBP}\right]}_{0}\right)}^{2}+4K_{d}C_{L}t}}{2{\left[\mathrm{GBP}\right]}_{0}}$$

where DE accounts for the differences in the detection efficiency of *L* and GBP ions.

COIN‐nMS has been used to inform on the glycan specificities of a variety of lectin targets, including plant lectins extensively used in lectin microarrays and tissue staining, immune lectins (e.g., C‐type lectins, Siglecs and galectins) and viral proteins (SARS‐CoV‐2 receptor binding domain, RBD), as well as to evaluate the affinities of CMP‐sialic acid inhibitors for sialyltransferases (Bui et al. 2023; Lin et al. 2023; Kumawat et al. 2024; Gray and Labasan et al. 2025). For example, COIN‐CaR‐nMS screening of natural glycan libraries against the plant lectin is *Ricinus communis* Agglutinin I (RCA‐I), which is commonly used to identify galactose (Gal) or *N*‐acetylgalactosamine (GalNAc) residues (Bojar et al. 2022), uncovered that nongalactosylated ligands with terminal N‐acetylglucosamine (GlcNAc) residues are also recognized with moderate to high affinity (Bui et al. 2023). Screening natural libraries of *N*‐glycans against the SARS‐CoV‐2 RBD revealed that sialylated *N*‐glycans were also ligands with comparable affinity as known ganglioside ligands (Nguyen et al. 2022; Bui et al. 2023).

The development of COIN‐CaR‐nMS (and CaR‐nMS) represents a major advance in shotgun glycomics. However, glycan ligand detection (and quantification) requires that the glycan be released from the GBP as an ion. Because glycans are relatively acidic in the gas phase (have low gas‐phase acidities) compared to the GBP ions, this requirement is generally satisfied by performing the measurements in negative ion mode (Park et al. 2020; Bui et al. 2022a, 2022c). However, it has been reported that sulfated glycan ligands (e.g., glycosaminoglycans, GAGs) are difficult to release, presumably due to unusually high gas‐phase stabilities of the GBP‐glycan complexes (Nguyen et al. 2022). Strategies that overcome this outstanding challenge are currently under development.

## 5 nMS Analysis of GBP–GL Interactions

### 5.1 nMS Studies of GBP–GL Interactions Using Model Membranes

**Figure 15:**
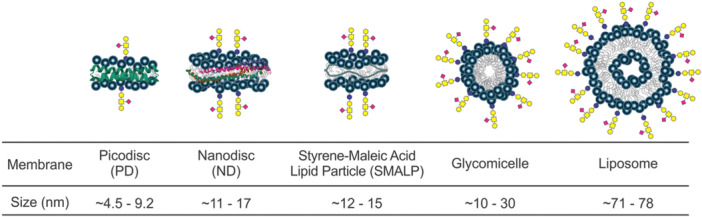
Overview of model membranes (PD, ND, SMALP, glycomicelle, and liposome) and their dimensions (effective diameter) that have been used for nMS‐based measurements of GBP–GL interactions. [Color figure can be viewed at wileyonlinelibrary.com]

Interactions between GBPs and cell surface GLs (e.g., glycosphingolipids [GSLs]) are involved in a number of biological and pathophysiological processes, such as the cellular adhesion and signaling, cell‐cell communication and immunological response (Lingwood et al. 2010; Schnaar et al. 2022). GLs also serve as the cellular receptors for bacterial and viral pathogens (Aerts et al. 2019; Nguyen et al. 2022). But, because available analytical methods have deficiencies, the vast majority of their interactions have yet to be identified and quantified. Glycolipids are amphipathic molecules with a hydrophilic oligosaccharide head group (which protrudes into the aqueous environment, making it accessible to GBPs) linked to a lipid moiety (e.g., ceramide) embeded into the cellular membrane. Due to the relatively insoluble nature of GLs and the importance of membrane environment on the thermodynamics of GBP–GL interactions, binding studies are typically implemented using model membranes displaying GLs. The model membranes assist in the solubilizing the GLs and provide a native‐like environment (Evans and Roger MacKenzie 1999; Lingwood et al. 2010; Nagafuku et al. 2003). There are three main classes of model membranes: (i) planar supported lipid bilayers; (ii) monolayer (e.g., micelles) and bilayer (e.g., liposomes) vesicles; and (iii) bilayer islands (e.g., nanodiscs [NDs], picodiscs [PDs], and styrene maleic acid lipid particles [SMALPs]) (Chan and Boxer 2007). Among these, mono‐ and bilayer vesicles and bilayer islands are generally compatible with nMS‐based binding assays. And GLs, alone (which spontaneously assemble into glycomicelles in aqueous solution at concentrations above the critical micelle concentration), or incorporated into liposomes, NDs, PDs and SMALPs (Figure 15) were utilized to detect and quantify GBP–GL interactions through nMS measurements (Han et al. 2022).

**Figure 16:**
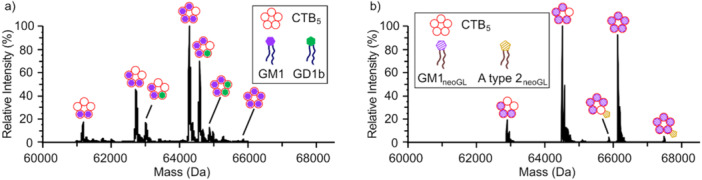
Deconvoluted nanoESI mass spectra acquired for solutions of CTB_5_ and NDs prepared with (a) the gangliosides GM1 and GD1b, and (b) neoglycolipids containing the oligosaccharidies of GM1 (GM1_neoGL_) and A type 2 blood group antigen (A type 2_neoGL_). The occupancy of the CTB_5_ primary and secondary binding sites corresponding to the detected ions is shown pictorially. Figures adapted from Han et al. 2020a and Han et al. 2020b. [Color figure can be viewed at wileyonlinelibrary.com]

Both nMS and CaR‐nMS have been used to detect GL interactions with GBPs. As noted above (Section 2.3.3), GBP–GL complexes detach from the membrane upon transfer to the gas phase during ESI due to the Coulombic repulsion (Han, Kitova, Li et al. 2015). The gaseous GBP–GL complex ions are kinetically stable in the gas phase and can be directly detected by nMS. This enables the identification of GL ligands present in the model membrane and provides a measure of the ligand binding stoichiometry (Han, Kitova, Li et al. 2015). For example, direct nMS measurements performed on solutions of CTB_5_ and a variety GM1‐containing model membranes have successfully detected CTB_5_ bound to up to five GM1 (Han et al. 2022). Other systems investigated include a fragment of family 51 carbohydrate‐binding module (CBM) binding with synthetic histo‐blood group type A and type B neoglycolipids (neoGLs), and GAL‐3C with gangliosides (Favell et al. 2024; Han et al. 2020b, 2015). Direct nMS measurements performed on CTB_5_ and NDs prepared with mixtures of GSLs or neoGLs provided the first molecular level evidence for hetero‐multivalent binding to GBPs (Figure 16) (Han et al. 2020b,; Heggelund et al. 2016). Moreover, affinity measurements performed using the proxy ligand nMS assay, vide infra, revealed that the binding of a high‐affinity ligand (e.g., GM1) dramatically enhanced, in some cases by three‐orders of magnitude, the binding of low‐affinity ligands present in the membrane (e.g., GD1b).

Glycolipid binding to GBPs can also be established, indirectly, using CaR‐nMS. This approach, which relies on the detection of GL ligands (as deprotonated ions) released from GBP complexes through CID, exhibits higher sensitivity than direct nMS and is able to detect weak (*K*_d_~1 mM) interactions. It is also more amenable to the analysis of heterogenous GBPs. For example, it has been used to demonstrate that SARS‐CoV‐2 RBD recognizes mono‐ and disialyated gangliosides displayed in NDs (Nguyen et al. 2022). This approach is also amenable to screening libraries of GLs in model membranes (defined GL mixtures or natural libraries of GLs extracted from tissue or cell cultures) against GBPs (Leney et al. 2014; Li et al. 2016).

Because the GBP and GBP–GL complex ions detected by nMS exhibit different ionization efficiencies (due to their distinct pathways to the gas phase), together with the possibility of incomplete extraction of GBP–GSL complexes (from the membrane) during ESI, their relative abundances measured in the gas phase do not necessarily reflect solution composition (Han et al. 2022). As a result of these nonuniform RFs, direct nMS analysis is generally unreliable for measuring GL binding affinities. For this reason, most nMS studies have been performed using the proxy ligand nMS assay (Section 2.3.3). To implement, a soluble oligosaccharide, which competes with the GL for GBP, serves as L_proxy_. This method is suitable for moderate to high affinities GBP–GL interactions, with *K*_d_ in the range of 0.1–100 µM. In the case of multivalent GBP–GL interactions, the experiments are generally designed so as to minimize the contribution from multivalency. This is done by using L_proxy_ concentrations that lead to, on average, to a single unoccupied binding site, thereby allowing for the intrinsic (per binding site) *K*_d_ value to be measured (Han et al. 2022; Nguyen et al. 2025).

The proxy ligand nMS assay has been used to measure the intrinsic affinity of GM1 in different model membranes for CTB_5_, to quantify hetero‐multivalent binding of CTB_5_ to low‐affinity gangliosides (in the presence of GM1) in NDs, and to evaluate the strength of binding for CBM and histo‐blood group type A and type B neoGLs (Han, Kitova, Li et al. 2015; Han et al. 2022). The assay has also been exploited to evaluate the effects of the membrane composition on GBP–GL interactions. For example, it was demonstrated that GL affinities are relatively insensitive to the structures of the membrane phospholipids or the presence of cholesterol (Han et al. 2017). In agreement with earlier studies (Hooper 1998; Shi et al. 2007), the affinities typically decrease (*K*_d_ increases) with increasing GL densities, a phenomenon attributed to clustering of GL in the membrane (resulting from H‐bonding between the oligosaccharide moieties) (Han et al. 2017). In addition, by comparing the natural GSL and neoGLs binding to GBP, it was shown that lipid moiety plays an insignificant role in GBP binding, raising the possibility of using synthetic neoGLs as surrogates for GSLs to accelerate the discovery of biologically relevant GBP–GL interactions (Han, Xue et al. 2020). The assay was also used to evaluate the influence of the model membrane on the strength of GBP binding with GLs (Han et al. 2022). Notably, it was found that the intrinsic affinity of CTB_5_ for GM1 incorporated into PDs, NDs, and SMALPs was comparable to those of the corresponding oligosaccharides. In contrast, the affinities measured using liposomes and glycomicelles were ∼10‐fold and ∼35‐fold weaker, an effect that was attributed to clustering of the GM1 within the membranes (Han et al. 2022). This finding highlighted the importance of the choice of model membrane used to quantify GBP–GL binding.

With the recent development of SLOMO‐nMS, which allows the relative *RF*s to be measured, it is now possible to directly quantify monovalent GBP binding to GLs in model membranes (Bui et al. 2022b). As an example, the *K*_d_ value for CBM binding to the B trisaccharide neoGL in an ND determined by SLOMO‐nMS (28.1 ± 3.0 μM) was found to be in good agreement with the value measured using the proxy ligand assay (20.2 ± 2 μM) (Han, Xue et al. 2020). SLOMO‐nMS also revealed that the relative *RF*s in such experiments can be significant (~25 to ~50), which is consistent with the operation of different pathways leading to the free GBP and GBP–GL complex ions. Efforts to adapt SLOMO‐nMS for quantifying multivalent GBP–GL binding are ongoing.

### 5.2 Membrane Anchor‐Assisted (MEAN) Native Mass Spectrometry

**Figure 17:**
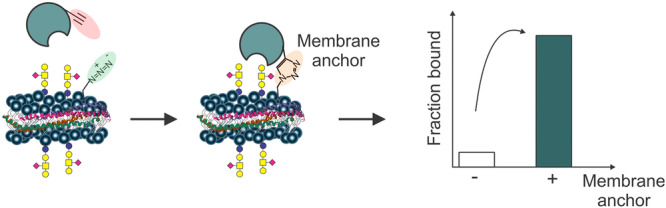
The MEmbrane ANchor‐assisted (MEAN)‐nMS assay for detecting GBP interactions with GLs in model membranes and cell membranes. The assay uses click chemistry to form membrane anchors that localize the labeled GBP to the surface of the membrane. This serves to increase the local concentration of GLs, leading to increased binding and facilitating the detection of GBP–GL complexes by nMS. [Color figure can be viewed at wileyonlinelibrary.com]

The low affinities of typical monovalent GBP−GL interactions challenge their detection by nMS. The MEmbrane ANchor‐assisted (MEAN)‐nMS assay—a recently developed method—employs a membrane anchor formed through click chemistry to enable the detection of low‐affinity interactions between soluble GBPs and GLs in model or cell membranes (Favell et al. 2024) (Figure 17). The membrane anchor is formed by labeling the GBP with alkyne‐containing groups (e.g., dibenzocyclooctyne [DBCO]) and reacting it with a membrane containing azide‐labeled phospholipid (e.g., azido phosphatidylethanolamine [azidoPE]). The copper‐free click reaction produces a covalent bond between the alkyne and the azide groups. The resulting membrane anchor, which localizes the GBP on the surface of the membrane, increases the effective GL concentration and, hence, promotes GBP–GL complex formation. Ligands are identified by nMS detection of intact GBP–GL complexes (MEAN‐nMS) or by releasing the GLs from GBP–GL complexes in the gas phase (MEAN‐CaR‐nMS).

The assay was validated by screening a library of purified gangliosides incorporated into NDs against two human galectins (GAL‐3C and GAL‐7), and comparing the results with the affinities of the corresponding ganglioside oligosaccharides. In the absence of a membrane anchor, nMS analysis yielded predominantly false negatives for these weak interactions. In contrast, all ganglioside ligands were identified by MEAN‐(CaR)‐nMS, with no false positives. Notably, the presence of the membrane anchor increased the fraction of bound GBP > 10 fold, which translates to an increase in effective GL concentration of two to three orders of magnitude. To highlight the potential of MEAN‐CaR‐nMS for ligand discovery, a natural library of gangliosides was incorporated into NDs and screened against human (Siglec‐1) and viral (SARS‐CoV‐2 RBD) GBPs to uncover elusive ganglioside ligands.

While originally conceived for use with model membranes, MEAN‐CaR‐nMS enabled, for the first time, nMS‐based detection of GBP of GSL ligands from intact cells. These preliminary data showcase the tremendous potential of MEAN‐(CaR)‐nMS, when implemented with a membrane anchor strategy, for discovering GSL ligands directly from cell membranes. This breakthrough sets the stage for implementing SG screening using intact cells.

## 6 Conclusions and Perspectives

Glycans play essential roles in diverse physiological and pathological processes through interactions with GBPs. Accurately characterizing GBP–glycan complexes is crucial for understanding their biological functions and holds significant potential for advancing diagnostics and therapeutics. Native MS offers a sensitive, label‐, and immobilization‐free method to directly analyze GBP–glycan interactions. With its ability to determine binding stoichiometry, affinities, thermodynamic parameters, and study multivalent and cooperative binding, nMS has greatly enhanced our understanding of these interactions across biological systems. Innovative methods such as SLOMO‐nMS make quantitative studies of GBP binding to glycoprotein ligands now possible. The development of MEAN‐nMS, which integrates click chemistry with nMS, has significantly enhanced the detection of low‐affinity GBP–GL interactions in model membranes and enables the discovery of GL ligands directly from intact cells, something previously not possible. Additionally, the establishment of COIN‐nMS introduces the potential for quantitative high‐throughput screening of natural glycan libraries without requiring precise concentration measurements.

Implementation of nMS for GBP–glycan binding studies is not without challenges, however. Factors such as changes in solution composition during nanoESI, in‐source dissociation, nonuniform response factors, and nonspecific binding require careful experimental design to ensure reliable results. Recent methodological advancements have addressed many of these issues. For example, submicron emitters minimize the occurrence of nonspecific binding. In cases where nonspecific binding can't be fully eliminated, the reference protein method can be applied to quantitatively correct mass spectra for these false positives. The SLOMO‐nMS approach resolves the problem of nonuniform *RF*s, and the proxy methods enable quantification of interactions that are otherwise undetectable or unresolved. Despite this tremendous progress, additional improvements are necessary to unlock the full potential of nMS. For example, enhanced instrumental performance is required for quantitative binding studies of large, heavily glycosylated GBPs. Furthermore, improved methods are needed to preserve and detect complexes of GBPs with mono‐ and disaccharides, which are difficult and often impossible to detect by nMS due to their tendency to dissociate in the ion source. It should also be noted that, in addition to nMS‐based methods, other MS approaches are emerging for the discovery and quantification of GBP‐glycan interactions. For example, glycan‐GBP cross‐linking and proximity labeling MS techniques have been developed to reveal cell‐surface glycan‐mediated protein interaction networks (Xie et al. 2021; Chen et al. 2025; Joeh et al. 2021). While matrix‐assisted laser desorption/ionization (MALDI) MS approaches, with and without chemical cross‐linking, for measuring glycosylation effects on protein‐glycoprotein affinities (Zhou et al. 2024) and *N*‐glycoprotein biomarker discovery (Black et al. 2019) have been described.

In summary, the emergence of nMS has greatly accelerated the discovery and characterization of GBP–glycan interactions. From defining immune lectin specificities to identifying novel glycan ligands for plant, bacterial, and viral GBPs, nMS is now an indispensable tool for uncovering the molecular basis of GBP–glycan recognition. Continued innovation in instrumentation and methodologies will further expand the applications of nMS in glycoscience, enabling deeper insights into the structure, function, and interactions of glycans in biological systems.

## Author Contributions

**Duong T. Bui:** conceptualization, writing – original draft, writing – review and editing. **John S. Klassen:** conceptualization, writing – original draft, writing – review and editing, supervision. **Elena N. Kitova:** writing – original draft, writing – review and editing. **Ling Han:** writing – review and editing. **Lara K. Mahal:** writing – review and editing, supervision.

## Conflicts of Interest

The authors declare no conflicts of interest.
